# A comparative study of adjuvants effects on neonatal plasma cell survival niche in bone marrow and persistence of humoral immune responses

**DOI:** 10.3389/fimmu.2022.904415

**Published:** 2022-08-03

**Authors:** Audur Anna Aradottir Pind, Sigrun Thorsdottir, Gudbjorg Julia Magnusdottir, Andreas Meinke, Giuseppe Del Giudice, Ingileif Jonsdottir, Stefania P. Bjarnarson

**Affiliations:** ^1^ Department of Immunology, Landspitali, The National University Hospital of Iceland, Reykjavik, Iceland; ^2^ Faculty of Medicine, School of Health Sciences, University of Iceland, Reykjavik, Iceland; ^3^ Valneva Austria GmbH, Vienna, Austria; ^4^ GSK Vaccines, Siena, Italy

**Keywords:** neonatal vaccination, adjuvant, comparative study, a proliferation inducing ligand (APRIL, TNFSF13), IL-6, B cell maturation antigen (BCMA, TNFRSF17), plasma cell survival niche

## Abstract

The neonatal immune system is distinct from the immune system of older individuals rendering neonates vulnerable to infections and poor responders to vaccination. Adjuvants can be used as tools to enhance immune responses to co-administered antigens. Antibody (Ab) persistence is mediated by long-lived plasma cells that reside in specialized survival niches in the bone marrow, and transient Ab responses in early life have been associated with decreased survival of plasma cells, possibly due to lack of survival factors. Various cells can secrete these factors and which cells are the main producers is still up for debate, especially in early life where this has not been fully addressed. The receptor BCMA and its ligand APRIL have been shown to be important in the maintenance of plasma cells and Abs. Herein, we assessed age-dependent maturation of a broad range of bone marrow accessory cells and their expression of the survival factors APRIL and IL-6. Furthermore, we performed a comparative analysis of the potential of 5 different adjuvants; LT-K63, mmCT, MF59, IC31 and alum, to enhance expression of survival factors and BCMA following immunization of neonatal mice with tetanus toxoid (TT) vaccine. We found that APRIL expression was reduced in the bone marrow of young mice whereas IL-6 expression was higher. Eosinophils, macrophages, megakaryocytes, monocytes and lymphocytes were important secretors of survival factors in early life but undefined cells also constituted a large fraction of secretors. Immunization and adjuvants enhanced APRIL expression but decreased IL-6 expression in bone marrow cells early after immunization. Furthermore, neonatal immunization with adjuvants enhanced the proportion of plasmablasts and plasma cells that expressed BCMA both in spleen and bone marrow. Enhanced BCMA expression correlated with enhanced vaccine-specific humoral responses, even though the effect of alum on BCMA was less pronounced than those of the other adjuvants at later time points. We propose that low APRIL expression in bone marrow as well as low BCMA expression of plasmablasts/plasma cells in early life together cause transient Ab responses and could represent targets to be triggered by vaccine adjuvants to induce persistent humoral immune responses in this age group.

## Introduction

The neonatal immune system is immature leaving neonates particularly vulnerable to infection and poor responders to vaccination. Low and transient antibody (Ab) responses following infection or vaccination in this age group have been associated with limited germinal center activation and decreased survival of plasma cells (1). In germinal centers, activated B cells undergo clonal expansion, affinity maturation, class switch recombination and can differentiate into memory B cells or plasmablasts that secrete Abs (2). Subsequently, plasmablasts can migrate to the bone marrow where they differentiate to long-lived plasma cells and persist (3). It has been reported that in neonatal mice that of the few plasmablasts formed in germinal centers, most of them home efficiently to the bone marrow but cannot persist due to lack of a proliferation inducing ligand (APRIL) (4), a critical survival factor for plasma cells. B cell maturation antigen (BCMA) is a high affinity receptor for APRIL whereas transmembrane activator calcium modulator and cyclophilin ligand interactor (TACI) binds APRIL with lower affinity (reviewed in (5)). TACI-deficient mice have diminished numbers of plasma cells, both in spleen and bone marrow (6) whereas BCMA-deficient mice display a drastic reduction in numbers of bone marrow plasma cells (7–9), leaving plasma cells in secondary lymphoid organs unaffected (8, 10) suggesting that BCMA is essential for survival of long-lived plasma cells in the bone marrow whereas TACI may be more important for the induction and survival of plasma cells in secondary lymphoid organs (reviewed in (11)).

A large fraction of vaccine candidates undergoing clinical development are made of highly purified recombinant protein or peptide antigens. This has driven the need for adjuvants as key components in modern vaccines since purified protein vaccines are rarely immunogenic (12). Adjuvants are immune-stimulating agents that can enhance and modulate responses to antigens and can be used as tools to enhance responsiveness to vaccines in vulnerable populations such as young infants (13). However, alum is the only adjuvant licensed for use in the pediatric population with the exception of MF59 and AS03 that have been licensed for use in seasonal and pandemic influenza vaccines (14). Thus, there is an unmet need for novel adjuvants and elucidation of their and other established adjuvants’ mechanisms of action in order to identify adjuvants active in early life and optimize vaccination responses in the pediatric population.

We evaluated effects of four adjuvants to overcome limitations of neonatal immunity and induce potent and persistent immune responses following neonatal immunization with the protein vaccine tetanus toxoid (TT) and compared with the previously established effect of LT-K63 (15). The adjuvants assessed are of various categories and have been reported to employ different mechanisms of action. We assessed effects of two toxin-based adjuvants, LT-K63, a mutant of *E.coli* heat labile enterotoxin and mmCT, a multiple mutant of cholera toxin (CT) derived from *V.cholarae*. MF59 is a squalene-based oil-in-water emulsion and has been licensed for use in children from 6 months of age (14). IC31 is a TLR9 agonist combined with an antimicrobial peptide (16) and lastly alum, the most widely used adjuvant that has been licensed in several paediatric vaccines. **Table 1** lists the adjuvants assessed herein and their main properties on adult and neonatal immune responses. We have previously compared the effects of the selected adjuvants with another vaccine, a pneumococcal conjugate Pnc1-TT, where we found that LT-K63, mmCT, MF59, and IC31, but not alum, enhanced germinal center formation and follicular dendritic cell maturation in neonatal mice which was associated with enhanced and prolonged persistence of vaccine-specific antibody-secreting cells (ASCs) and Abs up to 9 weeks after immunization (19, 20). However, alum only transiently enhanced vaccine-specific ASCs in bone marrow and serum Abs up to week 6 (20). Ab persistence is mediated by long-lived plasma cells that reside in specialized survival niches in the bone marrow (34) and their survival was recently shown to be dependent on direct contacts with stromal cells as well as APRIL : BCMA binding (35). In line with that, we demonstrated that LT-K63 enhanced early APRIL expression by bone marrow accessory cells, particularly by eosinophils, macrophages and megakaryocytes after immunization of neonatal mice with Pnc1-TT (15). Additionally, a higher proportion of plasmablasts and plasma cells of neonatal mice immunized with Pnc1-TT with LT-K63 expressed BCMA (15). Therefore, we wanted to explore whether the difference we previously observed (20) in the persistence of humoral immune responses induced by the selected adjuvants could be explained by their different effects on expression of plasma cell survival factors by bone marrow accessory cells and BCMA expression of plasmablasts/plasma cells up to this 6 week time point, where LT-K63 is used as a positive control. Furthermore, we assessed how the observed effects related to germinal center activation and induction of humoral immune responses. Prior to assessing the effects of neonatal immunization and adjuvants, we investigated age-dependent maturation of accessory cells of the plasma cell survival niche and their expression of survival factors APRIL and IL-6, for the first time to our best knowledge.

**Table 1:** Adjuvants assessed and their reported immune profiles.

Adjuvant	Composition	Immune profile	Early life immune profile
**LT-K63**	Toxin-based - mutant of LT	Th1 (17)	Th1/Th2 (18), Abs, FDCs, GCs (19), TNF-R and ligands (15), persistent ASC and Abs (19, 20)
**mmCT**	Toxin-based – mutant of CT	Th17 (21), IgA and IgG Abs (22, 23)	FDCs, GCs, persistent ASC and Abs (20)
**MF59^®^**	Squalene-based oil-in-water emulsion	Mixed Th1/Th2 (24) or Th2 and Abs (25)	Tfh (26), FDCs, GCs, persistent ASC and Abs (20)
**IC31^®^**	KLKL_5_KLK antimicrobial peptide with a synthetic TLR-9 agonist ODN1a	Mixed Th1/Th2 (16) or Th1 and Abs (27)	Mixed Th1/Th2 (28) or Th1 (29), Tfh cell (30), FDC, GC, persistent ASC and Abs (20)
**Alum**	Inorganic insoluble aluminum salts (aluminum hydroxide)	Th2 and Abs (31, 32)	Th2 and Abs (33)

LT, E.coli heat-labile enterotoxin; CT, Cholera toxin; FDC, follicular dendritic cell; GC, germinal center; TNF-R, tumor necrosis factor receptor; ASC, antibody-secreting cell; Abs, antibodies; Tfh, T follicular helper cell; TLR, Toll-like receptor.

## Materials and methods

### Mice

Adult NMRI mice were purchased from Taconic (Skansved, Denmark) and adapted for a minimum of one week after arrival before initiation experiments. For breeding of neonatal mice, two adult female mice were put in the cage of one adult male mouse for two weeks. Female mice were then separated from the male and kept in separate breeding cages which were checked daily for new births and the pups stayed with the mother until weaning at the age of 4 weeks. Mice were housed under standardized conditions at the vivarium facility Arctic Las (Reykjavík, Iceland) with regulated daylight, humidity and temperature and kept in micro-isolator cages where they had free access to commercial pelleted food and water. All experiments were carried out in accordance with Act No. 55/2013 on animal welfare and regulations 460/2017 on protection of animals used for scientific research. The protocol was approved by the Experimental Animal Committee of Iceland (license no. 2015-10-01).

### Vaccine, adjuvants, and immunization

Purified tetanus toxoid (TT) was purchased from Statens Serum Institute (Copenhagen, Denmark). LT-K63 (36) and MF59 (37) were produced and purified by Novartis Vaccines & Diagnostics (now GSK vaccines, Siena, Italy). mmCT was provided by Jan Holmgren, Michael Lebens and Manuela Terrinoni, from the Department of Microbiology and Immunology, Gothenburg University and was produced as described (22). IC31 was produced by Intercell AG, (now Valneva Austria GmbH, Vienna, Austria) as described (16). Aluminum hydroxide (Alhydrogel) was purchased from Brenntag Biosector A/S (Ballerup, Denmark). Neonatal (7 days old) mice were immunized with either vaccine alone, vaccine with adjuvant or saline as unimmunized controls. Mice were immunized subcutaneously (s.c.) at the base of the tail with 2 µg (0.8 limit of floculation, Lf) of purified TT (Statens Serum Institute) alone or mixed with the adjuvants LT-K63 (5 µg/mouse), mmCT (2 μg/mouse), MF59 (50% of injected volume/mouse), IC31 (50 nmol KLK and 2 nmol ODN1a/mouse) or alum (0.48% aluminum hydroxide per 1 μg of protein/mouse) in 50 μl of saline, or with 50 μl of saline alone as a control.

### Blood sampling

For blood collection, mice were bled from the tail vein and serum was prepared by centrifugation at 2400 rpm for 10 minutes at room temperature and stored at –20°C until use.

### Measurements of TT-specific antibodies in mouse sera

Measurement of TT-specific IgG antibodies was done using the following protocol. Microtiter plates (MaxiSorp) were coated with 5.0 µg/ml purified TT (Sanofi Pasteur) in 0.10 M carbonate buffer (pH 9.6) and incubated overnight at 4°C. Plates were washed 3 times with PBS containing 0.05% Tween 20 (v/v) (PBS-Tween20, Sigma) and then blocked with PBS-Tween20 containing 1% bovine serum albumin (BSA) for 1 hour at room temperature. Plates were washed as before and samples and standard were serially diluted (three-fold dilutions) and incubated in duplicates on TT-coated plates for 2 h at room temperature. The plates were washed as before and specific antibodies were detected with horseradish peroxidase (HRP)-conjugated goat anti-mouse antibody (Southern Biotechnology Associates Inc., Birmingham, AL, USA) diluted in PBS-Tween20 for 2 h at room temperature. As before the plates were washed and development of the enzyme reaction was performed by adding 100 µl of 3,3´,5,5´-tetramethylbenzidine peroxidase (TMB) substrate (Kirkegaard & Perry Laboratories, Gaithersburg, MD, USA) into each well for approximately 15 min and the reaction was stopped with 100 µl of 0.18 M H_2_SO_4_. The absorbance was read at 450 nm in a Titertek Multiscan Plus MK II spectrophotometer (ICN Flow Laboratories, Irvine, UK). Results were calculated from standard curves constructed by serial dilutions of a reference serum pool from hyperimmunized adult mice. The titers of the reference serum pool corresponded to the inverse serum dilution giving an optical density of 1.0, which has been assigned 100 ELISA units per ml (EU/ml). Results were expressed as mean log EU/ml ± standard deviation (SD).

### Measurements of TT-specific antibody-secreting cells in spleen and bone marrow

TT-specific ASC were enumerated by ELISPOT, as previously described (15, 19, 20, 38). MultiScreen High protein binding immobilon-P membrane plates (Millipore Corporation, Bedford, MA) were coated with 10 μg/ml TT overnight at 37°C, blocked with complete RPMI 1640 (Life Technologies BRL, Life Technologies, Paisley, U.K.). Duplicates of cells from spleen and bone marrow in four three-fold dilutions starting with 1 × 10^7^ cells in 100 μL in complete RPMI 1640 per well were incubated for 5 hours at 37°C, washed and incubated with ALP-goat anti-mouse IgG (Southern Biotechnology Associates) overnight at 4°C, and developed by 5-bromo-4-chloro-3-indolylphosphate and NBT in AP development buffer (Bio-Rad Labs, Hercules, CA). The number of spots, each representing a cell secreting TT-specific IgG antibodies, was counted with ELISPOT reader ImmunoSpot^®^ S6 ULTIMATE using ImmunoSpot^®^ SOFTWARE (Cellular Technology Limited (CTL) Europe, Bonn, Germany).

### Immunofluorescent staining of tissue sections

Spleens were frozen in Tissue-Tek OCT (Sakura, Zouterwoude, the Netherlands) and cut into 7 μm cryosections at 2 levels, starting 1,750 μm into the tissue and separated by 210 μm, fixed in acetone for 10 minutes, and stored at −70°C. Two sections per spleen (one from each level) were stained with fluorescent labeled IgM-FITC (BD Pharmingen) to visualize follicles, and biotinylated peanut agglutinin (PNA)-bio (Vector Laboratories, Burlingame, CA) to label dark-zone B cells, to visualize active GC reaction. Primary antibodies were incubated at room temperature for 30 minutes. The sections were then washed in PBS for 2 × 5 minutes prior to incubation with APC Streptavidin (BD Biosciences, Stockholm, Sweden) at room temperature for another 30 minutes and sections washed again as before. DAPI (Invitrogen, Eugene, OR) was used for nuclear counterstaining and incubated for the last 10 minutes of the later staining step. The sections were photographed with a digital camera (AXIOCAM; Zeiss) in a microscope (Zeiss) equipped with x10 and x40 objectives and AxioImaging Software (Birkerod, Denmark) for light and three-color immunofluorescence. Areas of PNA-positive staining were measured from all pictures using the AxioImaging Software.

### Immunofluorescence staining and flow cytometry

Spleens and bone marrow were collected 4, 8, 14 and 42 days after immunization for flow cytometry analysis using the following protocol as described (15). Single-cell suspensions from spleen and bone marrow were prepared and cells were washed and incubated (30 minutes on ice) in PBS with 0.5% BSA (Sigma) with 4 mmol/L EDTA (Sigma) with fluorochrome-labeled antibodies to B220, CD21, CD23, BAFF-R, CD138, Gr-1 (all from BD Biosciences), APRIL, CD11c, CD11b, CD200R3 (all from Biolegend), CD41, Siglec-F, F4/80 (all from eBioScience/Thermo Fisher) and BCMA (R&D Systems). Fc block (BD Biosciences), rat serum and mouse serum (2.7% each) was added to the staining mix to minimize unspecific binding. The stained cells were analyzed using Navios cytometer (Beckman Coulter, Brea, CA, USA) where recorded events were 400,000, and the generated data were analyzed by Kaluza^®^ analysis software (version 2.1 from Beckman Coulter) where dead cells and doublets were excluded prior to analysis.

### Statistical analysis

Mann-Whitney U test was used for comparison between groups and correlation was assessed using Spearman rank-order correlation applying a significance threshold of p<0.05 for both tests. All statistical analyses were carried out using Graphpad Prism 9.03 (GraphPad Software, La Jolla, CA).

## Results

### Limitations in APRIL, but not IL-6 expression in early life

**Figure 1:**
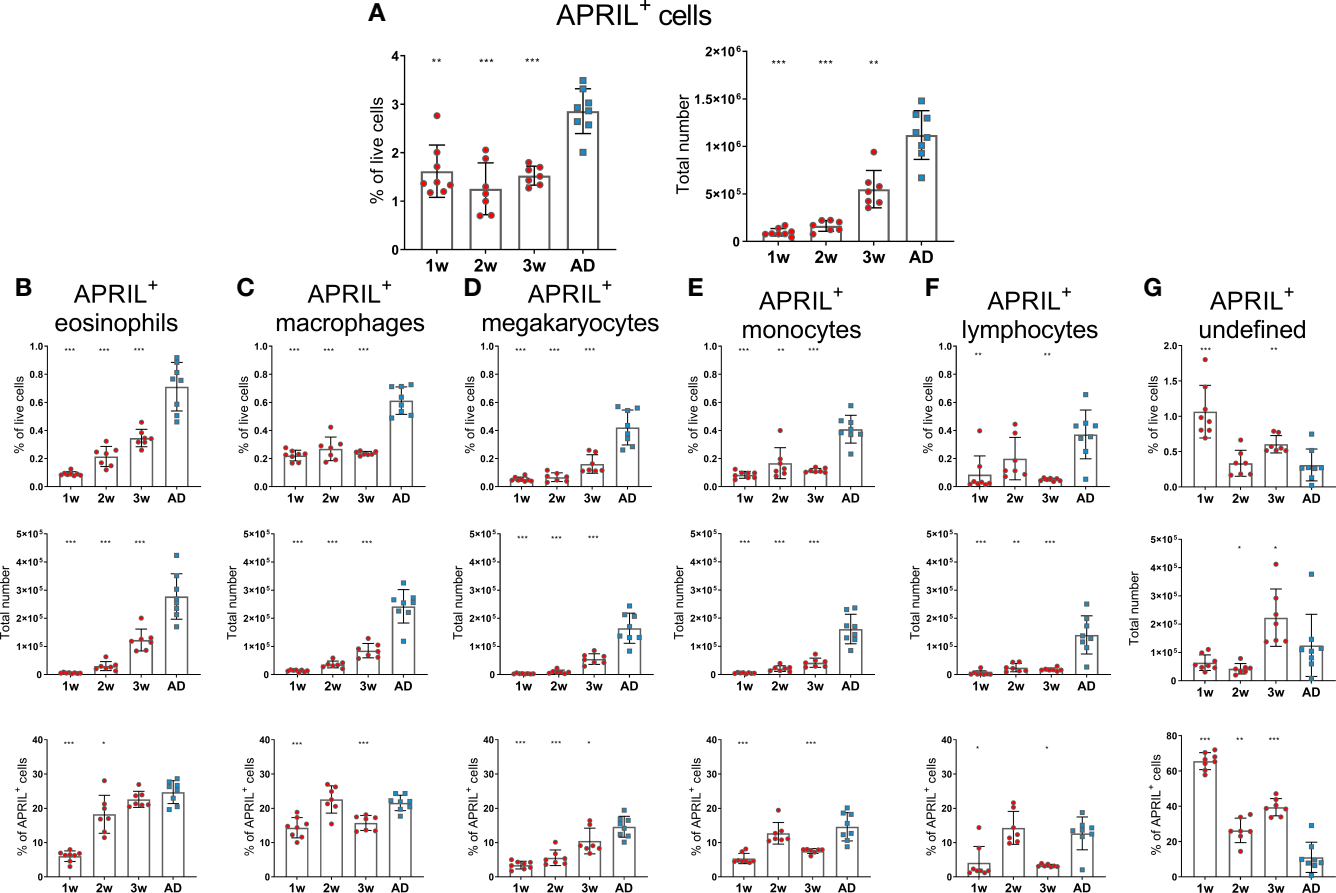
APRIL expression of bone marrow cells is limited in early life. Frequency and total number of APRIL^+^ cells **(A)** and frequency, total number and proportion out of total APRIL^+^ cells for APRIL^+^ eosinophils **(B)**, APRIL^+^ macrophages **(C)**, APRIL^+^ megakaryocytes **(D)**, APRIL^+^ monocytes **(E)**, APRIL^+^lymphcoytes **(F)** and undefined APRIL^+^ cells **(G)** in bone marrow assessed by flow cytometry in 1-, 2-, 3-week-old and adult (AD) mice. Each red circle and blue square represents one mouse and results are demonstrated as means ± SD. Mann Whitney U test was used for statistical comparison where values from 1-, 2- or 3-week-old-mice were compared to adult mice and *p ≤ 0.05, **p ≤ 0.01, ***p ≤ 0.001.

Before assessing the effects of neonatal immunization and adjuvants on bone marrow accessory cells and their expression of survival factors, we investigated their age-dependent maturation at steady state in 1-, 2-, 3-week-old and adult mice. In this study, we analyzed a broader range of accessory cell types than we had done previously (15). Eosinophils were defined as Gr-1^int^F4/80^+^CD11b^+^Siglec-F^+^SSC^high^, macrophages as Gr-1^+^F4/80^+^CD11b^+^Siglec-F^int^SSC^int^, megakaryocytes as CD41^+^F4/80^-^CD11c^-^Gr-1^-^FSC^high^, monocytes as Gr-1^int^F4/80^+^CD11b^+^Siglec-F^-^SSC^low^, basophils as CD200R3^+^F4/80^-/int^Gr-1^-/int^, neutrophils as Gr-1^+^F4/80^-^CD11b^+^ and dendritic cells as CD11c^+^CD200R^-^Siglec-F^-^ FSC^low^SSC^low^(**Supplementary Figure 1**). We found that APRIL expression by bone marrow cells was limited in early life as previously shown (4) and that it had not reached adult levels at 3 weeks of age (**Figure 1A**). Frequency and total number of eosinophils, megakaryocytes, monocytes and neutrophils were reduced in young mice and had not reached adult levels at 3 weeks of age (**Supplementary Figures 2A, C, D, F, G**). Macrophages and lymphocytes were limited in 1-2 week old mice, but reached adult levels at 3 weeks of age (**Supplementary Figures 2B, E**). When assessing APRIL expression of accessory cells we found that frequency and number of APRIL^+^ cells; eosinophils, macrophages, megakaryocytes, monocytes and lymphocytes was reduced in young mice and had not reached adult levels at 3 weeks of age (**Figures 1B–F**). Undefined APRIL^+^ cells, i.e. APRIL^+^ cells that did not fall into any of our flow cytometry gates, constituted for over 60% in 1 week old mice (**Figure 1G**). This high proportion of undefined cells among APRIL^+^ cells decreased with increasing age, and around 10% of APRIL^+^ cells remained undefined in adult mice (**Figure 1G**). Neutrophils, dendritic cells and basophils expressed very low levels of APRIL, constituting less than 1% of APRIL^+^ cells both in young or adult mice (data not shown).

**Figure 2:**
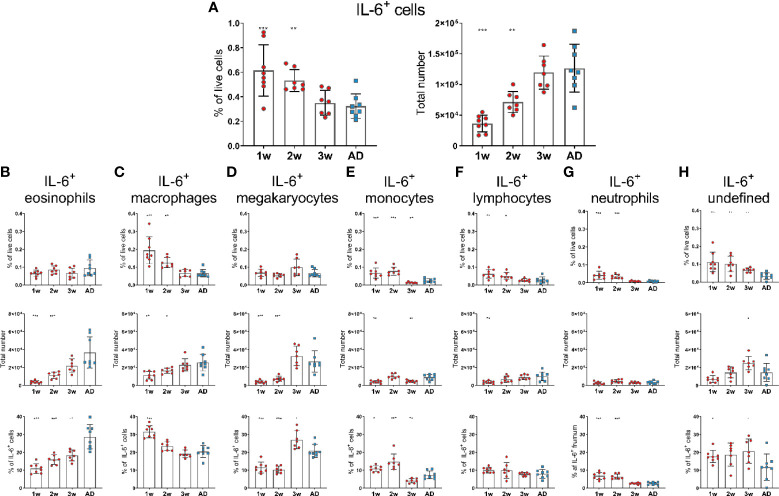
IL-6 expression of bone marrow cells is enhanced in early life. Frequency and total number of IL-6^+^ cells **(A)** and frequency, total number and proportion out of total IL-6^+^ cells for IL-6^+^eosinophils **(B)**, IL-6^+^ macrophages **(C)**, IL-6^+^ megakaryocytes **(D)**, IL-6^+^ monocytes **(E)**, IL-6^+^ lymphocytes **(F)**, IL-6^+^ neutrophils **(G)** and undefined IL-6^+^ cells **(H)** in bone marrow assessed by flow cytometry in 1-, 2-, 3-week-old and adult (AD) mice. Each red circle and blue square represents one mouse and results are demonstrated as means ± SD. Mann Whitney U test was used for statistical comparison where values from 1-, 2- or 3-week-oldmice were compared to adult mice and *p ≤ 0.05, **p ≤ 0.01, ***p ≤ 0.001.

In contrast to APRIL expression, IL-6 expression seemed to be higher in 1- and 2-week-old-mice than in adult mice, as they had increased frequency of IL-6^+^ cells. However, the total numbers of IL-6^+^ cells in 1- and 2-week-old-mice was still lower than in adult mice (**Figure 2A**). Frequency of IL-6^+^cells; macrophages, monocytes, lymphocytes and neutrophils, was higher in young mice than in adults (**Figures 2C, E, F, G**), whereas no difference was observed for eosinophils and megakaryocytes (**Figures 2B, D**). Undefined cells constituted for around 20% of IL-6^+^ cells in 1-, 2- and 3-week-old mice but had dropped down to 10% in adult mice (**Figure 2H**). Dendritic cells and basophils expressed very low levels of IL-6 and together constituted for less than 1% of IL-6^+^ cells, both in young and adult mice (data not shown).

Taken together, these data demonstrate that APRIL expression was limited in bone marrow at least up to 3 weeks of age whereas IL-6 expression was higher in 1-2-week-old than adult mice. Various accessory cells contributed to the production of these survival factors and undefined cells were more prominent at early age.

### Neonatal immunization and adjuvants enhance APRIL expression of bone marrow cells and BCMA expression of plasmablasts/plasma cells

**Figure 3:**
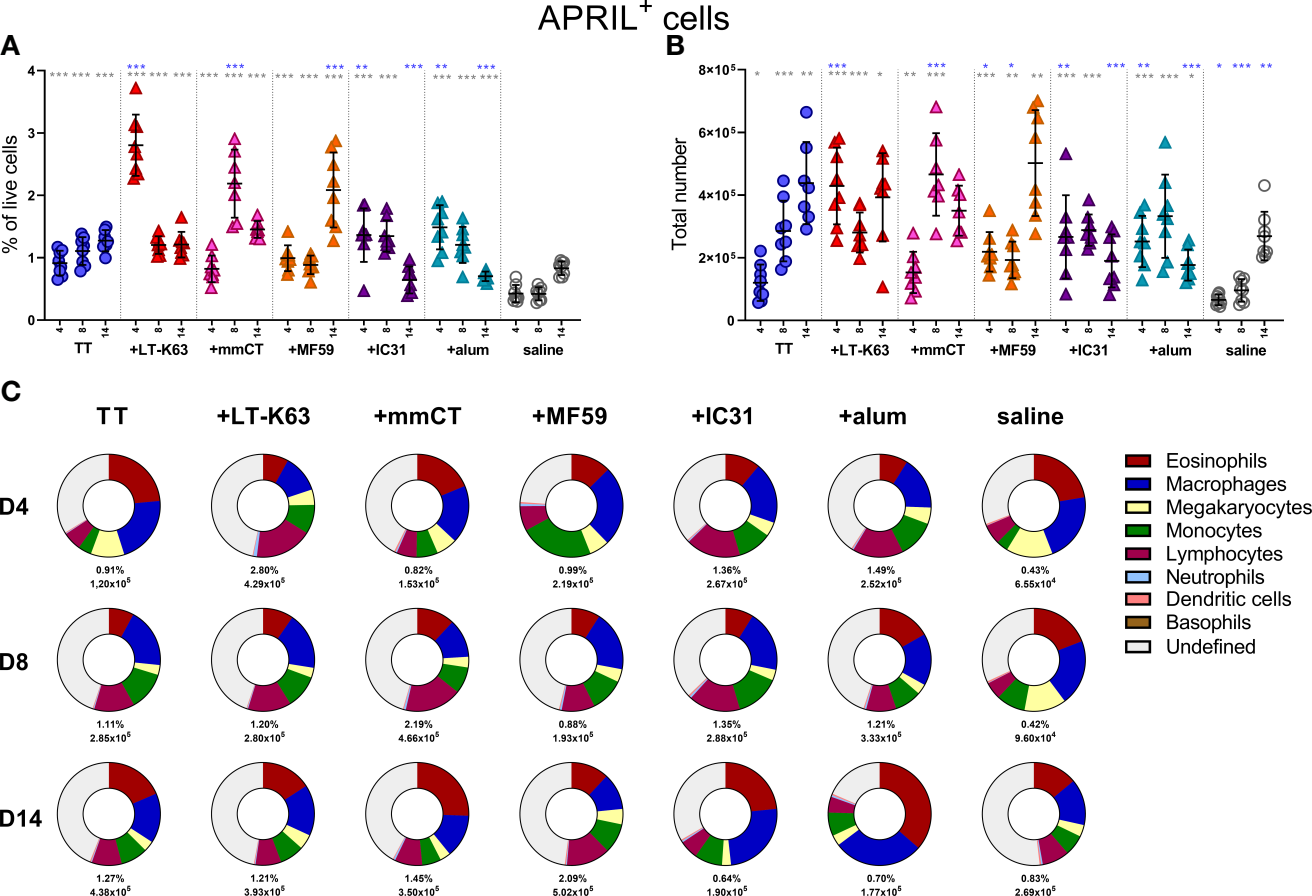
Neonatal immunization and adjuvants enhance APRIL expression in bone marrow cells. Frequency **(A)** and total numbers **(B)** of APRIL^+^ cells in bone marrow and mean distribution of APRIL^+^ cell types, mean frequencies and total numbers of APRIL^+^ cells for each group and time point **(C)** 4, 8 and 14 days after neonatal immunization with TT (blue circle) w/wo adjuvants LT-K63 (red triangle), mmCT (pink triangle), MF59 (orange triangle), IC31 (purple triangle), alum (turquoise triangle) or saline-injected mice (light grey circles) as controls. Each symbol represents one mouse and results are shown as means ± SD in 8 mice per group per time point (except n=7 for TT group on day 14 and n=7 for TT+mmCT group on days 8 and 14). For statistical evaluation Mann–Whitney U-test was used. Blue stars represent p values after comparison of TT group to adjuvant groups and grey stars represent comparisons of all the groups to saline group. *p ≤ 0.05, **p ≤ 0.01, ***p ≤ 0.001.

We have previously demonstrated that neonatal immunization with Pnc1-TT+LT-K63 enhanced APRIL expression of bone marrow cells early after immunization which associated with enhanced persistence of vaccine-specific humoral immune responses (15). We therefore wanted to address if the adjuvants tested also mediated their adjuvanticity through a similar mechanism. Thus, neonatal mice (7 days old) were immunized s.c. at base of tail with TT w/wo the adjuvants LT-K63, mmCT, MF59, IC31 or alum or injected with saline as controls. Immunization with TT alone enhanced both frequency and total numbers of APRIL^+^ cells in bone marrow when compared with saline-injected mice 4, 8 and 14 days after immunization (**Figures 3A, B** and **Supplementary Table 1**). Additionally, all the adjuvants assessed further enhanced APRIL^+^ cells in bone marrow, however with different kinetics (**Figures 3A, B**). LT-K63, IC31 and alum enhanced frequency and total number of APRIL^+^ cells 4 days after immunization, where the effect of LT-K63 was most pronounced. mmCT enhanced frequency and total number of APRIL^+^ cells 8 days after immunization and lastly, MF59 enhanced frequency of APRIL^+^ cells in bone marrow 14 days after immunization. Eosinophils, macrophages, monocytes and lymphocytes constituted considerable fractions of APRIL^+^ cells, but undefined cells were generally still the most abundant (**Figure 3C** and **Supplementary Table 2**). Of note, lymphocytes accounted for a large proportion of APRIL^+^ cells at the peak of its expression for each adjuvant (**Figure 3** and **Supplementary Table 2**).

When assessing the effect of immunization and adjuvants on the kinetics of accessory cell populations, we found that LT-K63, MF59, IC31 and alum enhanced megakaryocytes in bone marrow 4 days after immunization (**Supplementary Figure 3C**). Additionally, mmCT, MF59 and IC31 enhanced monocytes (**Supplementary Figure 3D**) and neutrophils (**Supplementary Figure 3G**) early after immunization, where neutrophils accounted for up to 30% of bone marrow cells in the TT+mmCT group 4 days after immunization (**Supplementary Figure 3G**). The kinetics of different APRIL^+^ accessory cells and undefined APRIL^+^ cells in bone marrow following immunization with TT w/wo adjuvants or saline are depicted in **Supplementary Figures 4A–F**.

**Figure 4:**
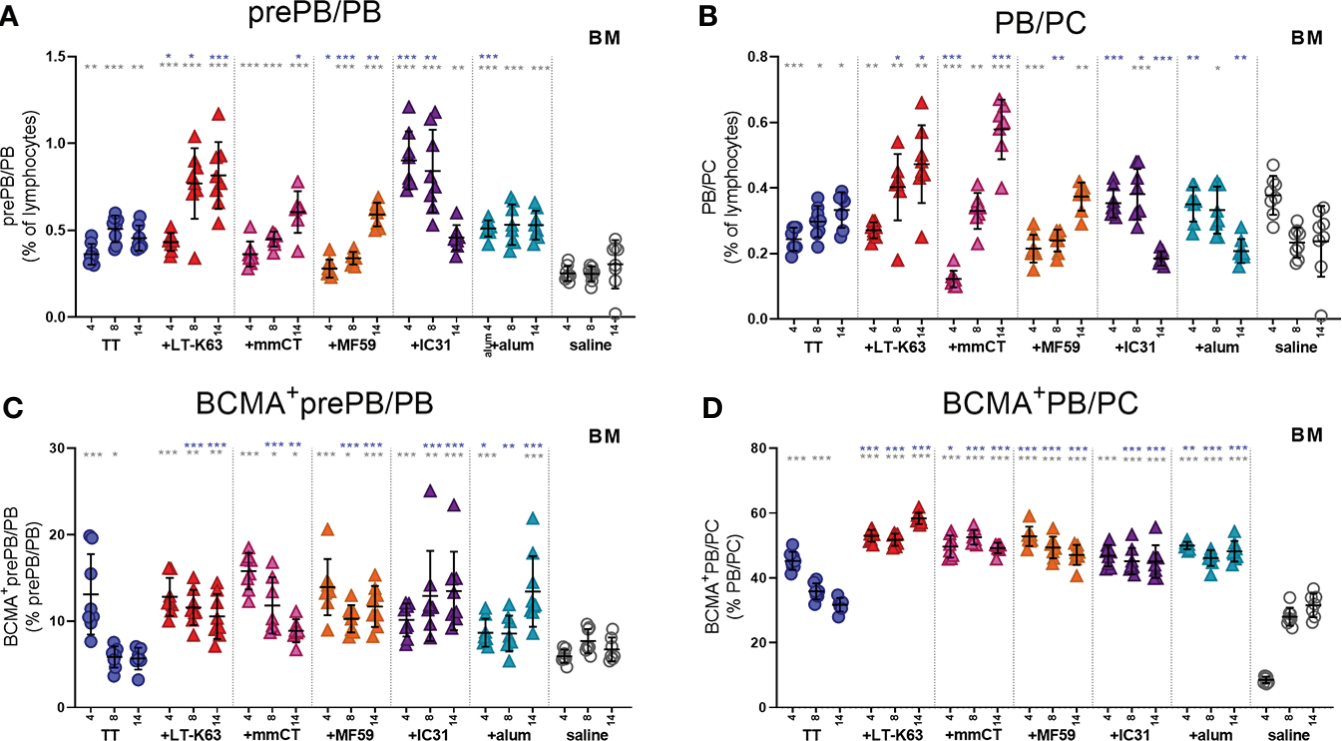
Neonatal immunization and adjuvants enhance early BCMA expression of plasmablasts/plasma cells in bone marrow. Frequency **(A, B)** and proportional BCMA expression **(C, D)** of B220^+^CD138^+^prePB/PB **(A, C)** and B220^+/-^CD138^high^ PB/PC **(B, D)** in bone marrow 4, 8 and 14 days after neonatal immunization with TT (blue circles) w/wo adjuvants LT-K63 (red triangle), mmCT (pink triangle), MF59 (orange triangle), IC31 (purple triangle), alum (turquoise triangle) or saline-injected mice (light grey circles) as controls. Each symbol represents one mouse and results are shown as means ± SD in 8 mice per group per time point (except n=7 for TT group on day 14 and n=7 for TT+mmCT group on days 8 and 14). For statistical evaluation Mann–Whitney U-test was used. Blue stars represent p values after comparison of TT group to adjuvant groups and grey stars represent comparisons of all the groups to saline group. *p ≤ 0.05, **p ≤ 0.01, ***p ≤ 0.001.

It was recently reported that both stromal cell contact and binding of APRIL to BCMA is required for plasma cell survival (35). Since APRIL expression was enhanced by bone marrow cells early after immunization we next assessed BCMA expression of bone marrow plasmablasts/plasma cells at these early time points. Like we had observed before (15), we found that CD138 expression was lower in neonatal than adult mice so two populations of plasmablasts/plasma cells were assessed. Firstly, we defined B220^+^CD138^+^ cells as pre-plasmablasts/plasmablasts (prePB/PB), containing both pre-plasmablasts and plasmablasts (4, 39) and secondly B220^+/-^CD138^high^ cells were defined as plasmablasts/plasma cells (PB/PC), containing both plasmablasts and plasma cells (39, 40). Immunization with TT alone subtly enhanced the proportion of bone marrow plasmablasts/plasma cells at these early time points (**Figures 4A, B**). Most of the adjuvants further enhanced plasmablasts/plasma cells but with different kinetics, where the effects of LT-K63 and IC31 were most pronounced (**Figures 4A, B** and **Supplementary Figures 5A, B**). Likewise, immunization with TT alone transiently enhanced the proportion of plasmablasts/plasma cells that expressed BCMA, whereas inclusion of each of the adjuvants further enhanced their proportion that persisted up to 14 days after immunization (**Figures 4C, D**). Of note, the immunization and adjuvant effects on the PB/PC subset were more pronounced as a much higher proportion of this subset was BCMA^+^ than of the prePB/PB subset in all immunization groups (**Figures 4C, D**).

Taken together, immunization and adjuvants enhanced both APRIL expression by accessory cells and BCMA expression of plasmablasts/plasma cells in bone marrow at early time points after immunization but the effects were more pronounced when adjuvants were included.

### Neonatal immunization and adjuvants decrease IL-6 expression by bone marrow cells

**Figure 5:**
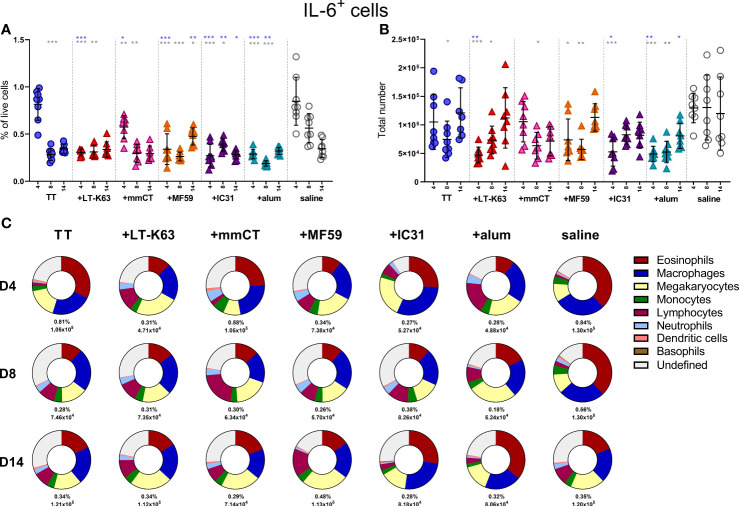
Neonatal immunization and adjuvants decrease IL-6 expression in bone marrow cells. Frequency **(A)** and total numbers **(B)** of IL-6^+^ cells in bone marrow and mean distribution of IL-6^+^ cell types, mean frequencies and total numbers of IL-6^+^ cells for each group and time point **(C)** 4, 8 and 14 days following neonatal immunization with TT (blue circle) w/wo adjuvants LT-K63 (red triangle), mmCT (pink triangle), MF59 (orange triangle), IC31 (purple triangle), alum (turquoise triangle) or saline-injected mice (light grey circles) as controls. Each symbol represents one mouse and results are shown as means ± SD in 8 mice per group per time point (except n=7 for TT group on day 14 and n=7 for TT+mmCT group on days 8 and 14). For statistical evaluation Mann–Whitney U-test was used. Blue stars represent p values after comparison of TT group to adjuvant groups and grey stars represent comparisons of all the groups to saline group. *p ≤ 0.05, **p ≤ 0.01, ***p ≤ 0.001.

Since we found that immunization and adjuvants enhanced APRIL^+^ cells in bone marrow early after immunization we were curious to know if the same effects would be observed for IL-6^+^ cells, considering that like APRIL, IL-6 has been linked to prolonged plasma cell survival (41–43). On the contrary, immunization with TT alone decreased IL-6^+^ cells in the bone marrow 8 days after immunization and including adjuvants in the immunization accelerated this decrease (**Figure 5A, B** and **Supplementary Table 3**). As for APRIL^+^ cells, eosinophils, macrophages and lymphocytes all constituted a considerable fraction of IL-6^+^ cells, but megakaryocytes and undefined cells were also abundant (**Figure 5C** and **Supplementary Table 4**). The kinetics of different IL-6^+^ accessory cells and undefined IL-6^+^ cells in bone marrow following immunization are depicted in **Supplementary Figure 6A–G**.

### Enhanced BCMA expression of splenic plasmablasts and plasma cells by adjuvants correlates with enhanced TT-specific antibody-secreting cells in spleen and serum antibodies 2 weeks after immunization

Next we wanted to assess if immunization and adjuvants mediated similar effects on splenic plasmablasts/plasma cells and their BCMA expression like we observed for bone marrow and if these effects could be connected to enhanced induction of vaccine-specific humoral responses.

**Figure 6:**
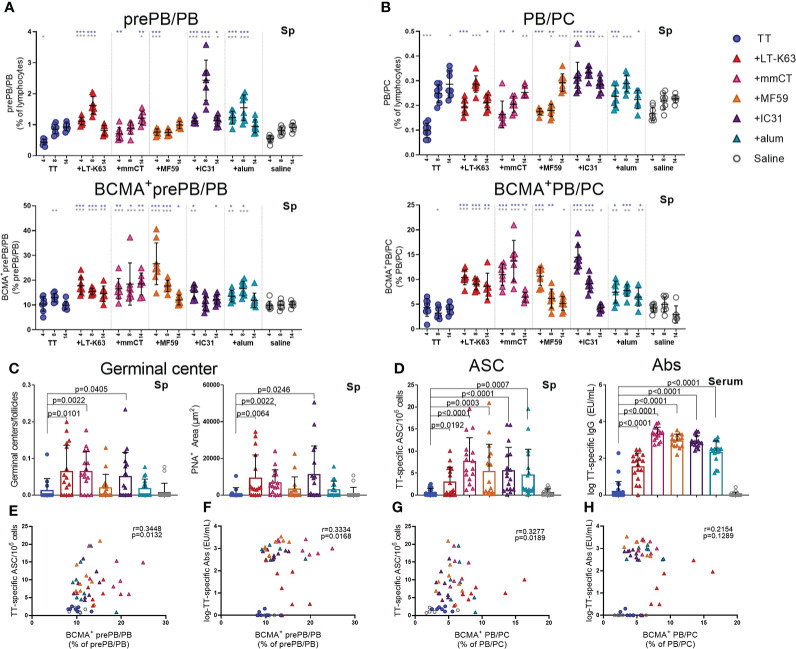
Adjuvants enhance BCMA expression of plasmablasts/plasma cells in spleen correlating with enhanced TT-specific antibody-secreting cells and antibodies. Frequency and BCMA expression of B220^+^CD138^+^prePB/PB **(A)** and B220^+/-^CD138^high^ PB/PC **(B)** in spleen 4, 8 and 14 days after neonatal immunization with TT (blue circles) w/wo adjuvants LT-K63 (red triangle), mmCT (pink triangle), MF59 (orange triangle), IC31 (purple triangle), alum (turquoise triangle) or saline-injected mice (light grey circles) as controls. Germinal center activation determined by fluorescent staining of spleen sections with PNA and anti-IgM 14 days after immunization of neonatal mice. PNA/IgM ratio represents activated GCs in relation to total number of follicles (**C**, left panel) and PNA^+^ area represents total area of positive PNA staining per section (**C** right panel). TT-specific antibody-secreting cells (ASC) in spleen (**D**, left panel) and TT-specific IgG serum antibodies (**D**, right panel) 14 days after immunization. Each symbol represents one mouse and results are shown as means ± SD in 8 mice per group per time point (except n=7 for TT group on day 14 and n=7 for TT+mmCT group on days 8 and 14). Results for germinal center induction **(C)**, ASCs and Abs **(D)** are pooled from two independent experiments. For statistical evaluation Mann–Whitney U-test was used. Blue stars represent p values after comparison of TT group to adjuvant groups and grey stars represent comparisons of all the groups to saline group. *p ≤ 0.05, **p ≤ 0.01, ***p ≤ 0.001. In C-H, p values are visible on the figures. Spearman correlation plots for evaluation of association between BCMA^+^prePB/PB frequency and TT-specific ASC **(E)** or TT-specific IgG Abs **(F)** or BCMA^+^ PB/PC frequency and TT-specific ASC **(G)** or TT-specific IgG Abs **(H)** 14 days after immunization.

Thus, neonatal mice were immunized as before and spleens harvested at various time points after immunization. We found that immunization with TT alone did not increase plasmablasts/plasma cells nor enhance their BCMA expression (**Figures 6A, B**, **Supplementary Figures 7A, B** and **Supplementary Table 6**). On the contrary, including adjuvants in the immunization enhanced both prePB/PB and PB/PC in spleen at early time points and the proportion BCMA^+^ cells (**Figures 6A, B**, **Supplementary Figure 7A, B**, **Supplementary Table 6**).

In response to a protein antigen, activated B cells can enter germinal center reactions where class switching, affinity maturation and differentiation into memory B cells or plasmablasts/plasma cells occurs (44). Germinal centers are generally attenuated in human infants (45–47), but some adjuvants have been shown to overcome limitations and induce potent germinal centers in early life murine models (19, 20, 30). We had previously demonstrated that the adjuvants LT-K63, mmCT, MF59 and IC31 enhanced germinal center induction after neonatal immunization with a pneumococcal conjugate vaccine (Pnc1-TT). In order to assess if the adjuvants would have similar effects on germinal center induction with a purified protein, TT vaccine, spleens were obtained 8 and 14 days after immunization of neonatal mice. We found that mmCT enhanced germinal center induction 8 and 14 days after immunization (**Figure 6C**, **Supplementary Figure 7D**) and LT-K63 and IC31 only at day 14 (**Figure 6C**). On the contrary, neither MF59 nor alum enhanced germinal center induction after neonatal immunization with TT. However, all adjuvants enhanced TT-specific IgG^+^ASCs in spleen (**Figure 6D**, left) and TT-specific IgG Abs in serum (**Figure 6D**, right) 14 days after immunization. Of note, the adjuvants mmCT, MF59 and IC31 additionally enhanced TT-specific ASC in bone marrow already at this same time point. Furthermore, the adjuvants LT-K63, mmCT and MF59 prolonged the induction in spleen since TT-specific IgG^+^ ASC were still enhanced 6 weeks after immunization (**Supplementary Figure 7C**). To assess if there was any association between BCMA expression of plasmablasts/plasma cells and vaccine-specific responses we analyzed the correlation between proportional BCMA expression of the two plasmablast/plasma cell populations and TT-specific ASC and Abs 14 days after immunization. A significant correlation was found between proportional BCMA expression of prePB/PB and TT-specific ASC (**Figure 6E**) and Abs (**Figure 6F**) and also between proportional BCMA expression of PB/PC and TT-specific ASC (**Figure 6G**) but not Abs (**Figure 6H**).

Taken together, all the adjuvants enhanced BCMA expression by plasmablasts and plasma cells in spleen, which correlated with enhanced vaccine-specific humoral immune responses. However, only LT-K63, mmCT and IC31 enhanced GC induction.

### Enhanced BCMA expression of bone marrow plasmablasts and plasma cells by adjuvants correlates with vaccine-specific humoral immune responses 6 weeks after immunization

**Figure 7:**
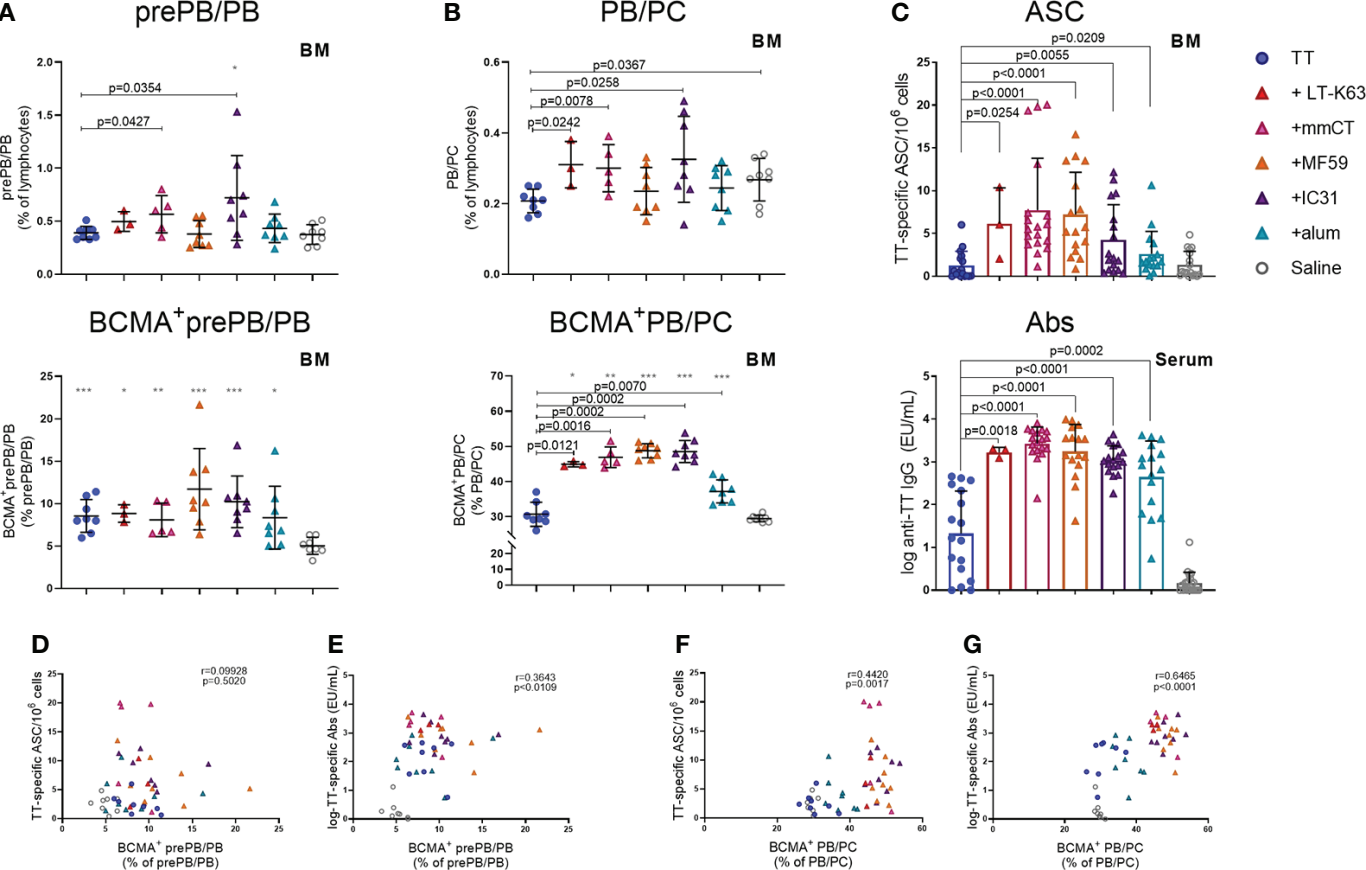
Adjuvants enhance BCMA expression of plasmablasts/plasma cells correlating with enhanced vaccine-specific humoral responses 6 weeks after immunization. Frequency (upper panels) and proportional BCMA expression (lower panels) of B220^+^CD138^+^prePB/PB **(A)** and B220^+/-^CD138^high^ PB/PC **(B)**, TT-specific antibody-secreting cells (ASC) in bone marrow (**C**, upper panel) and TT-specific IgG serum antibodies (Abs) (**C**, lower panel) 6 weeks after neonatal immunization with TT (blue circles) w/wo adjuvants LT-K63 (red triangle), mmCT (pink triangle), MF59 (orange triangle), IC31 (purple triangle), alum (turquoise triangle) or saline-injected mice (light grey circles) as controls. Spearman correlation plots for evaluation of association between BCMA^+^prePB/PB frequency and TT-specific ASC **(D)** or TT-specific IgG Abs **(E)** or BCMA^+^ PB/PC frequency and TT-specific ASC **(F)** or TT-specific IgG Abs **(G)** 6 weeks after immunization. Each symbol represents one mouse and results are shown as means ± SD in 8 mice per group (except n=3 for TT+LT-K63 group n=5 for TT+mmCT group). Results for ASC and Abs **(C)** are pooled from two independent experiments. For statistical evaluation Mann–Whitney U-test was used. P values from comparisons between TT group and adjuvants groups are visible on the figures whereas grey stars represent comparisons of all groups to saline group. *p ≤ 0.05, **p ≤ 0.01, ***p ≤ 0.001.

Lastly, we wanted to explore if enhanced APRIL expression by bone marrow cells early after immunization and increased BCMA expression of plasmablasts/plasma cells by adjuvants were associated with enhanced persistence of vaccine-specific humoral immune responses. We were also curious to know if BCMA expression by plasmablasts/plasma cells induced by adjuvants persisted after immunization. Thus, we assessed the frequency and total numbers of plasmablasts/plasma cells and TT-specific ASC in bone marrow and TT-specific serum Abs 6 weeks after immunization. At this time point, we did not detect much effect of immunization and adjuvants on the frequency and BCMA expression of prePB/PB in bone marrow (**Figure 7A**, **Supplementary Table 5**). However, PB/PC of mice immunized as neonates with each of the adjuvants were more frequently BCMA^+^ than PB/PC of mice immunized with vaccine alone or saline-injected mice (**Figure 7B**). Of note, even though neonatal immunization with TT+alum enhanced BCMA expression by PB/PC to a higher degree than immunization with TT alone at this time point, LT-K63 (p=0.0121), mmCT (p=0.0016), MF59 (p=0.0002) and IC31 (p=0.0002) were superior to alum in inducing persistent enhanced BCMA expression. All of the adjuvants enhanced TT-specific ASC in bone marrow and serum Abs at this time point (**Figure 7C**). Again, mmCT (p=0.0003 for ASC and p=0.0008 for Abs) and MF59 (p=0.0014 for ASC and p=0.0415 for Abs) were superior to alum in enhancing vaccine-specific ASC and Abs. To explore if there was any association between BCMA expression of plasmablasts/plasma cells in bone marrow and vaccine-specific responses, we assessed the correlation between proportional BCMA expression of the two plasmablast/plasma cell populations and TT-specific ASC in bone marrow and serum Abs 6 weeks after immunization. A significant correlation was observed between proportional BCMA expression of prePB/PB and TT-specific Abs (**Figure 7E**) but not ASC (**Figure 7D**) and also between proportional BCMA expression of PB/PC and TT-specific ASC (**Figure 7F**) and Abs (**Figure 7G**). Interestingly, mice immunized with the adjuvants LT-K63, mmCT, MF59 and IC31 grouped together in the correlation plot while alum-immunized mice rather grouped with mice immunized with TT alone (**Figure 7G**).

To summarize, immunization with adjuvants induced a higher proportion of PB/PC to express BCMA which persisted up to 6 weeks, although these effects were significantly less pronounced for alum. This enhanced BCMA expression correlated with persistent vaccine-specific humoral immune responses.

## Discussion

It has previously been reported from experiments using mouse models that the majority of plasmablasts emerging from germinal centers after neonatal and infant immunization efficiently migrates to the bone marrow (48). However, poor APRIL expression by bone marrow stromal cells (4) was associated with reduced persistence and enhanced apoptosis of plasmablasts (48). This lack of survival signals in neonatal bone marrow could therefore explain transient Ab responses reported in this age group (1), since Ab persistence is mediated by long-lived plasma cells that reside in specialized survival niches in the bone marrow (34). APRIL can be expressed by stromal cells but can additionally be expressed by various hematopoietic cells, also termed accessory cells, in the bone marrow (49, 50). For the first time in a neonatal mouse model we performed a comparative analysis of different adjuvants on APRIL expression by accessory cells in the bone marrow. Before assessing effects of immunization and different adjuvants, we wanted to assess, also for the first time, age-dependent maturation of bone marrow accessory cells and their expression of the plasma cell survival factors APRIL and IL-6 at steady state. We found that APRIL was poorly expressed by early life accessory cells, but this was not the case for IL-6. Furthermore, neonatal immunization, in particular with adjuvants, enhanced APRIL expression by accessory cells but decreased their IL-6 expression. This is in line with what we previously showed for LT-K63, when administered with a pneumococcal conjugate vaccine (15) (and unpublished data).

Importantly, all five adjuvants assessed herein enhanced APRIL expression in bone marrow but the extent and kinetics of this APRIL enhancement differed between adjuvants. Early increase in APRIL following immunization was not expected since plasma cell influx to the bone marrow after immunization usually remains low until after more than 3 weeks (19). However, soluble APRIL can be bound by heparan sulphate proteoglycans (51, 52) in the bone marrow and APRIL bound to proteoglycan has been found to be superior to soluble APRIL in activation of B cells (52, 53). In line with that, APRIL-rich niches with plasma cells have been found in human mucosa where heparan sulphate proteoglycans retained neutrophil-derived APRIL (54). Furthermore, mice deficient in glucuronyl C5-epimerase, an enzyme that controls heparan sulphate chain flexibility affecting ligand binding, failed to respond to APRIL-mediated survival signals resulting in reduced plasma cells and Ab levels (55), emphasizing the importance of heparan sulphate proteoglycans in plasma cell survival.

It must be noted that the effects of adjuvants on APRIL expression was less pronounced than we had previously reported when neonatal mice were immunized with Pnc1-TT with LT-K63 (15). However, there were some fundamental differences between the sets of experiments described herein and the experiments previously published (15). In the previous study T cells, B cells, NK cells, dendritic cells, mast cells and basophils were depleted from the bone marrow cell suspension prior to analysing APRIL expression and accessory cells. Therefore, the cells that have potential of APRIL expression might have been more concentrated in the earlier study. Additionally, the depletion protocol might have stimulated the cells to some extent. Finally, the cells were analysed in a different flow cytometer and thus inevitably with different settings.

It was surprising that immunization and adjuvants decreased IL-6 expression by bone marrow cells since IL-6 has been identified as a plasma cell survival factor (41–43). Nonetheless, other data from mice suggest that IL-6 seems to only be required for induction but not maintenance of plasma cells *in vivo (* 56). Additionally, blocking of IL-6R using tocilizumab has not been shown to affect serum IgG Ab levels of patients (57). Of note, co-injection of adult mice with IL-6 and a pneumococcal conjugate vaccine enhanced T follicular helper (Tfh) cells and T follicular regulatory (Tfr) cells that was associated with improved Ab responses. On the contrary, the same immunization protocol in neonatal mice reduced the expansion of Tfh cells but increased Tfr cells that led to limited Ab responses. This could be explained by enhanced expression of IL-6R by neonatal compared with adult Tfr cells and decreased IL-6R expression of neonatal Tfh cells compared with adult Tfh cells (58). This suggests that even though IL-6 can be beneficial for induction of immune responses in adult setting it could have opposite effects in a neonatal setting.

How the adjuvants induce their effects on cells of the bone marrow is still unclear. We find it unlikely that the adjuvants reach the bone marrow to directly activate cells there, although we cannot exclude that possibility. A more plausible explanation would be that they induce influx of immune cells to site of injection, and through the engagement of pattern recognition receptors or other receptors lead to activation and secretion of pro-inflammatory cytokines and chemokines that in turn enhances hematopoiesis and activation of cells in the bone marrow. During infection or inflammation, hematopoietic stem cells respond to inflammatory stimuli by emergency myelopoiesis (59). Interestingly, enhanced IL-6 was recently found to be involved in age-associated hematopoietic decline (60). Therefore, decreased IL-6 expression in bone marrow cells early after immunization, which was more pronounced with the inclusion of adjuvants, could be a sign of enhanced hematopoiesis (**Figure 5A**).

A large fraction of both APRIL^+^ cells and IL-6^+^ cells in bone marrow remained undefined, i.e. didn’t fall into any of our assigned cell population gates. These cells accounted for more than 60% of APRIL^+^ cells in 1 week old mice but decreased with increasing age. Thus, they may represent precursor cells and therefore lack efficient expression of cell-identifying surface markers. Interestingly, bone marrow neutrophil precursors have been shown to express APRIL in adult mice (61). Another explanation may be that some other cell types that were not assessed herein expressed plasma cell survival factors to a higher degree in younger than adult mice. Like before (15), eosinophils, macrophages and megakaryocytes constituted a big part of APRIL^+^ cells in bone marrow but herein we additionally found that monocytes and lymphocytes were frequently APRIL^+^. Likewise, eosinophils, macrophages, megakaryocytes and lymphocytes constituted a big fraction of IL-6^+^ cells.

BCMA is predominantly expressed by GC B cells, memory B cells and plasma cells (5) and has been shown to be needed for survival of long-lived plasma cells in the bone marrow, as BCMA-deficiency in mice drastically reduced numbers of bone marrow plasma cells (7–9), leaving plasma cells in secondary lymphoid organs unaffected (8, 10). Following differentiation of B cells into plasmablasts in secondary lymphoid organs they can relocate to the bone marrow, and it has been demonstrated that plasmablasts sufficiently migrate to the early life bone marrow compartments following neonatal immunization (48). Like we had previously found for LT-K63 (15) when administered with a pneumococcal conjugate vaccine, immunization with TT, and in particular when adjuvants were included, enhanced BCMA expression of plasmablasts/plasma cells, both in spleen and bone marrow. This enhanced BCMA expression of the PB/PC subset induced by adjuvants was still observed 6 weeks after immunization. Enhanced BCMA expression of plasma cells may render them more fit for prolonged survival, enabling binding of APRIL. BCMA expression of plasmablasts/plasma cells correlated with vaccine-specific ASCs, early in spleen and later in bone marrow, and with serum Abs. The correlation was generally weaker in the spleen and the best correlation was observed between proportional BCMA expression of PB/PC subset with TT-specific Abs 6 weeks after immunization. This indicates a strong association between BCMA expression and Abs at later time points after immunization, fitting well with previous publications demonstrating that BCMA is essential for long-lived plasma cells (8).

In our previous work (20) we demonstrated that LT-K63, mmCT, MF59 and IC31 but not alum accelerated maturation of follicular dendritic cells and enhanced germinal center induction when they were administered to neonatal mice with a pneumococcal conjugate vaccine, Pnc1-TT. Herein, only LT-K63, mmCT and IC31 enhanced germinal center induction, but MF59 and alum did not. Even though MF59 and alum did not enhance germinal center formation, both adjuvants induced enhanced TT-specific IgG^+^ ASC in spleen and IgG serum Abs at 2 and 6 weeks after immunization, compared with TT alone. Of interest, MF59 could enhance IgG Abs following immunization of CD4 knockout mice or CD4-depleted mice after immunization with a TD influenza virus split vaccine (62) suggesting that a CD4-independent pathway bypassing GC induction can be an alternative mechanism for MF59. It might be that the timepoints assessed were suboptimal for assessing GC induction following immunization with TT, since vaccine adjuvants have been shown to differently affect kinetics of germinal center responses (63), or it could be that these adjuvants trigger more extra-follicular responses. However, the adjuvanticity of MF59 has been shown to be mediated through enhanced Tfh cells and in turn enhanced germinal center induction, but MF59 was unable to activate Tfh cells following neonatal immunization with HA (26).

We have also previously shown that a single immunization of neonatal mice with Pnc1-TT with the adjuvants LT-K63, mmCT, MF59 and IC31, but not alum, was sufficient to induce vaccine-specific ASCs in bone marrow and serum Abs that persisted above protective levels against pneumococcal bacteremia and lung infection 9 weeks after immunization (20). On the contrary, alum only transiently enhanced vaccine-specific ASCs in bone marrow and serum Abs up to week 6 (20). In this study, all the adjuvants induced higher levels of IgG Abs than TT alone. Of interest, bone marrow PB/PC of mice immunized with TT+alum were less frequently BCMA^+^ 6 weeks after immunization than PB/PC of mice immunized with any of the other adjuvants assessed herein and may therefore be less fit for prolonged survival as was observed in previous studies (20, 64).

It was surprising that adjuvants with previously established different mechanisms of action (**Table 1**) all induced similar responses in our model, i.e. enhanced APRIL and BCMA expression, although with different kinetics and magnitudes and decreased IL-6 expression, that associated with enhanced humoral immune responses. It still remains unknown through which mechanisms this enhanced APRIL and BCMA expression and decreased IL-6 expression is mediated and likely they differ between adjuvants, and will be studied in more detail in future experiments. Nonetheless, a significant correlation of BCMA expression among plasmablasts/plasma cells and serum Abs 6 weeks after immunization suggests that upregulation of BCMA on plasmablasts/plasma cells is an important step in rendering these cells more fit for prolonged survival and induction of persistent Ab responses after neonatal immunization. In line with that, mice immunized with LT-K63, mmCT, MF59 and IC31 clearly grouped together on this correlation plot whereas mice immunized with alum grouped with mice immunized with TT alone (**Figure 7G**), revealing differences between alum and the other adjuvants that could explain more transient responses induced by alum as previously reported by us and others (20, 64).

To summarize, we found that APRIL expression was limited in young mice whereas IL-6 expression was higher in younger than adult mice. We identified eosinophils, macrophages, megakaryocytes, monocytes and lymphocytes as important secretors of survival factors in early life but undefined cells also constituted a large fraction of secretors. Neonatal immunization and adjuvants enhanced APRIL expression but decreased IL-6 expression in bone marrow cells early after immunization. Moreover, immunization and adjuvants enhanced proportions of plasmablasts/plasma cells that expressed BCMA early in spleen and later in bone marrow, and this enhanced BCMA expression significantly correlated with enhanced vaccine-specific humoral responses. It must be noted that alum’s effect on BCMA expression was less pronounced at later time points than the effects of the other adjuvants which could explain previous reports of transient humoral immune responses induced by alum (20, 64). We demonstrate that not only APRIL is limited in early life, but also BCMA expression of plasmablasts/plasma cells and that enhanced BCMA expression induced by adjuvants correlated with enhanced persistence of vaccine-specific humoral immune responses, offering an explanation for transient Ab responses in early life. These results together with our previously published data (20) warrant further investigations of the adjuvants mmCT, MF59 and IC31 for use in early life vaccinology.

## Data availability statement

The raw data supporting the conclusions of this article will be made available by the authors, without undue reservation.

## Ethics statement

The animal study was carried out in accordance with Act No. 55/2013 on animal welfare and regulations 460/2017 on protection of animals used for scientific research. The protocol was reviewed and approved by Experimental Animal Committee of Iceland, MAST, Austurvegur 64, 800 Selfoss, Iceland (license no. 2015-10-01).

## Author contributions

AP, IJ, and SB conceived and designed the study, interpreted the results and wrote the manuscript. IJ and SB supervised the study. AP, GM, ST, and SB performed the experiments. AP and SB analyzed the data. AM and GD provided material and expertise. All authors contributed to and approved the final version of the manuscript.

## Funding

AP was a recipient of a doctoral study grant from the University of Iceland Research Fund (2015-18). This study was financially supported by grants from the Icelandic Research Fund (130675051-53), The University of Iceland Research Fund (2018-20) and the Landspitali Science Fund (A-2017-068, A-2017-069, A-2018-076, A-2018-077, A-2019-084).

## Acknowledgments

We thank Professor Jan Holmgren, MD, PhD, Michael Lebens, PhD and Manuela Terrinoni at the Department of Microbiology and Immunology at University of Gothenburg, for providing the adjuvant mmCT and Professor Jan Holmgren for his expert advice. Part of the work presented in this paper was presented as a poster at the 50^th^ Anniversary Meeting of the Scandinavian Society for Immunology, Aarhus, October 19^th^-22^nd^ 2021 and in an oral presentation on the 48^th^ Annual Meeting of the Scandinavian Society for Immunology, Reykjavík, June 12^th^-15^th^ 2022.

## Conflict of interest

GD is a previous employee and holds shares in the GSK group of companies. AM is an employee of Valneva Austria GmbH.

The remaining authors declare that the research was conducted in the absence of any commercial or financial relationships that could be construed as a potential conflict of interest.

## Publisher’s note

All claims expressed in this article are solely those of the authors and do not necessarily represent those of their affiliated organizations, or those of the publisher, the editors and the reviewers. Any product that may be evaluated in this article, or claim that may be made by its manufacturer, is not guaranteed or endorsed by the publisher.

## References

[1] Siegrist CA Aspinall R. B-cell responses to vaccination at the extremes of age. Nat Rev Immunol (2009) 9:185–94. doi: 10.1038/nri2508 19240757

[2] Huang C. Germinal center reaction. Adv Exp Med Biol (2020) 1254:47–53. doi: 10.1007/978-981-15-3532-1_4 32323268

[3] Radbruch A Muehlinghaus G Luger EO Inamine A Smith KG Dorner T. Competence and competition: The challenge of becoming a long-lived plasma cell. Nat Rev Immunol (2006) 6:741–50. doi: 10.1038/nri1886 16977339

[4] Belnoue E Pihlgren M McGaha TL Tougne C Rochat AF Bossen C. APRIL is critical for plasmablast survival in the bone marrow and poorly expressed by early-life bone marrow stromal cells. Blood (2008) 111:2755–64. doi: 10.1182/blood-2007-09-110858 18180376

[5] Dostert C Grusdat M Letellier E Brenner D. The TNF family of ligands and receptors: Communication modules in the immune system and beyond. Physiol Rev (2019) 99:115–60. doi: 10.1152/physrev.00045.2017 30354964

[6] Ou X Xu S Lam K-P. Deficiency in TNFRSF13B (TACI) expands T-follicular helper and germinal center b cells *via* increased ICOS-ligand expression but impairs plasma cell survival. Proc Natl Acad Sci (2012) 109:15401–6. doi: 10.1073/pnas.1200386109 PMC3458353 22949644

[7] Peperzak V Vikstrom I Walker J Glaser SP LePage M Coquery CM. Mcl-1 is essential for the survival of plasma cells. Nat Immunol (2013) 14:290–7. doi: 10.1038/ni.2527 PMC4041127 23377201

[8] O'Connor BP Raman VS Erickson LD Cook WJ Weaver LK Ahonen C. BCMA is essential for the survival of long-lived bone marrow plasma cells. J Exp Med (2004) 199:91–8. doi: 10.1084/jem.20031330 PMC1887725 14707116

[9] Benson MJ Dillon SR Castigli E Geha RS Xu S Lam KP. Cutting edge: the dependence of plasma cells and independence of memory b cells on BAFF and APRIL. J Immunol (Baltimore Md 1950) (2008) 180:3655–9. doi: 10.4049/jimmunol.180.6.3655 18322170

[10] Xu S Lam KP. B-cell maturation protein, which binds the tumor necrosis factor family members BAFF and APRIL, is dispensable for humoral immune responses. Mol Cell Biol (2001) 21:4067–74. doi: 10.1128/mcb.21.12.4067-4074.2001 PMC87068 11359913

[11] Cornelis R Chang HD Radbruch A. Keeping up with the stress of antibody production: BAFF and APRIL maintain memory plasma cells. Curr Opin Immunol (2021) 71:97–102. doi: 10.1016/j.coi.2021.06.012 34303157

[12] Del Giudice G Rappuoli R Didierlaurent AM. Correlates of adjuvanticity: A review on adjuvants in licensed vaccines. Semin Immunol (2018) 39:14–21. doi: 10.1016/j.smim.2018.05.001 29801750

[13] Nanishi E Dowling DJ Levy O. Toward precision adjuvants: optimizing science and safety. Curr Opin Pediatr (2020) 32:125–38. doi: 10.1097/mop.0000000000000868 PMC6970548 31904601

[14] Wilkins AL Kazmin D Napolitani G Clutterbuck EA Pulendra B Siegrist CA. AS03- and MF59-adjuvanted influenza vaccines in children. Front Immunol (2017) 8:1760. doi: 10.3389/fimmu.2017.01760 29326687 PMC5733358

[15] Aradottir Pind AA Molina Estupiñan JL Magnusdottir GJ Del Giudice G Jonsdottir I Bjarnarson SP. LT-K63 enhances b cell activation and survival factors in neonatal mice that translates into long-lived humoral immunity. Front Immunol (2020) 11:527310. doi: 10.3389/fimmu.2020.527310 33193301 PMC7644473

[16] Schellack C Prinz K Egyed A Fritz JH Wittmann B Ginzler M. IC31, a novel adjuvant signaling *via* TLR9, induces potent cellular and humoral immune responses. Vaccine (2006) 24:5461–72. doi: 10.1016/j.vaccine.2006.03.071 16678312

[17] Ryan EJ McNeela E Pizza M Rappuoli R O'Neill L Mills KH. Modulation of innate and acquired immune responses by escherichia coli heat-labile toxin: Distinct pro- and anti-inflammatory effects of the nontoxic AB complex and the enzyme activity. J Immunol (Baltimore Md 1950) (2000) 165:5750–9. doi: 10.4049/jimmunol.165.10.5750 11067933

[18] Olafsdottir TA Hannesdottir SG Giudice GD Trannoy E Jonsdottir I. Effects of LT-K63 and CpG2006 on phenotype and function of murine neonatal lymphoid cells. Scandinavian J Immunol (2007) 66:426–34. doi: 10.1111/j.1365-3083.2007.01970.x 17850587

[19] Bjarnarson SP Adarna BC Benonisson H Del Giudice G Jonsdottir I. The adjuvant LT-K63 can restore delayed maturation of follicular dendritic cells and poor persistence of both protein- and polysaccharide-specific antibody-secreting cells in neonatal mice. J Immunol (Baltimore Md 1950) (2012) 189:1265–73. doi: 10.4049/jimmunol.1200761 PMC3496199 22753937

[20] Aradottir Pind AA Dubik M Thorsdottir S Meinke A Harandi AM Holmgren J. Adjuvants enhance the induction of germinal center and antibody secreting cells in spleen and their persistence in bone marrow of neonatal mice. Front Immunol (2019) 10:2214. doi: 10.3389/fimmu.2019.02214 31616417 PMC6775194

[21] Larena M Holmgren J Lebens M Terrinoni M Lundgren A. Cholera toxin, and the related nontoxic adjuvants mmCT and dmLT, promote human Th17 responses *via* cyclic AMP-protein kinase a and inflammasome-dependent IL-1 signaling. J Immunol (Baltimore Md 1950) (2015) 194:3829–39. doi: 10.4049/jimmunol.1401633 25786687

[22] Lebens M Terrinoni M Karlsson SL Larena M Gustafsson-Hedberg T Kallgard S. Construction and preclinical evaluation of mmCT, a novel mutant cholera toxin adjuvant that can be efficiently produced in genetically manipulated vibrio cholerae. Vaccine (2016) 34:2121–8. doi: 10.1016/j.vaccine.2016.03.002 26973069

[23] Terrinoni M Holmgren J Lebens M Larena M. Requirement for cyclic AMP/Protein kinase a-dependent canonical NFkappaB signaling in the adjuvant action of cholera toxin and its non-toxic derivative mmCT. Front Immunol (2019) 10:269. doi: 10.3389/fimmu.2019.00269 30838003 PMC6389712

[24] Vono M Taccone M Caccin P Gallotta M Donvito G Falzoni S. The adjuvant MF59 induces ATP release from muscle that potentiates response to vaccination. Proc Natl Acad Sci USA (2013) 110:21095–100. doi: 10.1073/pnas.1319784110 PMC3876261 24324152

[25] Podda A Del Giudice G. MF59-adjuvanted vaccines: increased immunogenicity with an optimal safety profile. Expert Rev Vaccines (2003) 2:197–203. doi: 10.1586/14760584.2.2.197 12899571

[26] Mastelic Gavillet B Eberhardt CS Auderset F Castellino F Seubert A Tregoning JS. MF59 mediates its b cell adjuvanticity by promoting T follicular helper cells and thus germinal center responses in adult and early life. J Immunol (Baltimore Md 1950) (2015) 194:4836–45. doi: 10.4049/jimmunol.1402071 25870238

[27] Knudsen NP. Different human vaccine adjuvants promote distinct antigen-independent immunological signatures tailored to different pathogens. Sci Rep (2016) 6:19570. doi: 10.1038/srep19570 26791076 PMC4726129

[28] Olafsdottir TA Lingnau K Nagy E Jonsdottir I. Novel protein-based pneumococcal vaccines administered with the Th1-promoting adjuvant IC31 induce protective immunity against pneumococcal disease in neonatal mice. Infection Immun (2012) 80:461–8. doi: 10.1128/iai.05801-11 PMC3255653 22025519

[29] Kamath AT Rochat AF Valenti MP Agger EM Lingnau K Andersen P. Adult-like anti-mycobacterial T cell and *in vivo* dendritic cell responses following neonatal immunization with Ag85B-ESAT-6 in the IC31 adjuvant. PLoS One (2008) 3:e3683. doi: 10.1371/journal.pone.0003683 18997860 PMC2577009

[30] Vono M Eberhardt CS Mohr E Auderset F Christensen D Schmolke M. Overcoming the neonatal limitations of inducing germinal centers through liposome-based adjuvants including c-type lectin agonists trehalose dibehenate or curdlan. Front Immunol (2018) 9:381. doi: 10.3389/fimmu.2018.00381 29541075 PMC5835515

[31] Brewer JM Conacher M Hunter CA Mohrs M Brombacher F Alexander J. Aluminium hydroxide adjuvant initiates strong antigen-specific Th2 responses in the absence of IL-4- or IL-13-mediated signaling. J Immunol (Baltimore Md 1950) (1999) 163:6448–54. 10586035

[32] Grun JL Maurer PH. Different T helper cell subsets elicited in mice utilizing two different adjuvant vehicles: The role of endogenous interleukin 1 in proliferative responses. Cell Immunol (1989) 121:134–45. doi: 10.1016/0008-8749(89)90011-7 2524278

[33] Dowling DJ Levy O. Pediatric vaccine adjuvants: Components of the modern vaccinologist's toolbox. Pediatr Infect Dis J (2015) 34:1395–8. doi: 10.1097/inf.0000000000000893 PMC4931280 26353029

[34] Chang HD Tokoyoda K Radbruch A. Immunological memories of the bone marrow. Immunol Rev (2018) 283:86–98. doi: 10.1111/imr.12656 29664564 PMC5947123

[35] Cornelis R Hahne S Taddeo A Petkau G Malko D Durek P. Stromal cell-contact dependent PI3K and APRIL induced NF-κB signaling prevent mitochondrial- and ER stress induced death of memory plasma cells. Cell Rep (2020) 32:107982. doi: 10.1016/j.celrep.2020.107982 32755576 PMC7408492

[36] Giuliani MM Del Giudice G Giannelli V Dougan G Douce G Rappuoli R. Mucosal adjuvanticity and immunogenicity of LTR72, a novel mutant of escherichia coli heat-labile enterotoxin with partial knockout of ADP-ribosyltransferase activity. J Exp Med (1998) 187:1123–32. doi: 10.1084/jem.187.7.1123 PMC2212201 9529328

[37] O'Hagan DT Ott GS Nest GV Rappuoli R Giudice GD. The history of MF59((R)) adjuvant: A phoenix that arose from the ashes. Expert Rev Vaccines (2013) 12:13–30. doi: 10.1586/erv.12.140 23256736

[38] Bjarnarson SP Benonisson H Del Giudice G Jonsdottir I. Pneumococcal polysaccharide abrogates conjugate-induced germinal center reaction and depletes antibody secreting cell pool, causing hyporesponsiveness. PloS One (2013) 8:e72588. doi: 10.1371/journal.pone.0072588 24069152 PMC3771989

[39] Wilmore JR Jones DD Allman D. Protocol for improved resolution of plasma cell subpopulations by flow cytometry. Eur J Immunol (2017) 47:1386–8. doi: 10.1002/eji.201746944 PMC5584378 28654161

[40] Pracht K Meinzinger J Daum P Schulz SR Reimer D Hauke M. A new staining protocol for detection of murine antibody-secreting plasma cell subsets by flow cytometry. Eur J Immunol (2017) 47:1389–92. doi: 10.1002/eji.201747019 28608550

[41] Chu VT Berek C. Immunization induces activation of bone marrow eosinophils required for plasma cell survival. Eur J Immunol (2012) 42:130–7. doi: 10.1002/eji.201141953 22057654

[42] Rodriguez Gomez M Talke Y Goebel N Hermann F Reich B Mack M. Basophils support the survival of plasma cells in mice. J Immunol (Baltimore Md 1950) (2010) 185:7180–5. doi: 10.4049/jimmunol.1002319 21068399

[43] Gabler J Wittmann J Porstner M Renz H Jäck HM Abram M. Contribution of microRNA 24-3p and Erk1/2 to interleukin-6-mediated plasma cell survival. Eur J Immunol (2013) 43:3028–37. doi: 10.1002/eji.201243271 23934711

[44] Young C Brink R. The unique biology of germinal center b cells. Immunity (2021) 54:1652–64. doi: 10.1016/j.immuni.2021.07.015 34380063

[45] Kruschinski C Zidan M Debertin AS von Hörsten S Pabst R. Age-dependent development of the splenic marginal zone in human infants is associated with different causes of death. Hum Pathol (2004) 35:113–21. doi: 10.1016/s0046-8177(03)00422-2 14745733

[46] Timens W Boes A Rozeboom-Uiterwijk T Poppema S. Immaturity of the human splenic marginal zone in infancy. Possible contribution to the deficient infant immune response. J Immunol (1989) 143:3200–6. 2478621

[47] Zandvoort A Lodewijk ME de Boer NK Dammers PM Kroese FG Timens W. CD27 expression in the human splenic marginal zone: The infant marginal zone is populated by naive b cells. Tissue Antigens (2001) 58:234–42. doi: 10.1034/j.1399-0039.2001.580403.x 11782274

[48] Pihlgren M Friedli M Tougne C Rochat AF Lambert PH Siegrist CA. Reduced ability of neonatal and early-life bone marrow stromal cells to support plasmablast survival. J Immunol (Baltimore Md 1950) (2006) 176:165–72. doi: 10.4049/jimmunol.176.1.165 16365407

[49] Chang HD Radbruch A. Maintenance of quiescent immune memory in the bone marrow. Eur J Immunol (2021) 51:1592–601. doi: 10.1002/eji.202049012 34010475

[50] Zehentmeier S Roth K Cseresnyes Z Sercan O Horn K Niesner RA. Static and dynamic components synergize to form a stable survival niche for bone marrow plasma cells. Eur J Immunol (2014) 44:2306–17. doi: 10.1002/eji.201344313 24777940

[51] Hendriks J Planelles L de Jong-Odding J Hardenberg G Pals ST Hahne M. Heparan sulfate proteoglycan binding promotes APRIL-induced tumor cell proliferation. Cell Death differentiation (2005) 12:637–48. doi: 10.1038/sj.cdd.4401647 15846369

[52] Ingold K Zumsteg A Tardivel A Huard B Steiner QG Cachero TG. Identification of proteoglycans as the APRIL-specific binding partners. J Exp Med (2005) 201:1375–83. doi: 10.1084/jem.20042309 PMC2213192 15851487

[53] Kimberley FC van Bostelen L Cameron K Hardenberg G Marquart JA Hahne M. The proteoglycan (heparan sulfate proteoglycan) binding domain of APRIL serves as a platform for ligand multimerization and cross-linking. FASEB J Off Publ Fed Am Societies Exp Biol (2009) 23:1584–95. doi: 10.1096/fj.08-124669 19141538

[54] Huard B McKee T Bosshard C Durual S Matthes T Myit S. APRIL secreted by neutrophils binds to heparan sulfate proteoglycans to create plasma cell niches in human mucosa. J Clin Invest (2008) 118:2887–95. doi: 10.1172/jci33760 PMC2447926 18618015

[55] Reijmers RM Groen RW Kuil A Weijer K Kimberley FC Medema JP. Disruption of heparan sulfate proteoglycan conformation perturbs b-cell maturation and APRIL-mediated plasma cell survival. Blood (2011) 117:6162–71. doi: 10.1182/blood-2010-12-325522 21471524

[56] Cassese G Arce S Hauser AE Lehnert K Moewes B Mostarac M. Plasma cell survival is mediated by synergistic effects of cytokines and adhesion-dependent signals. J Immunol (Baltimore Md 1950) (2003) 171:1684–90. doi: 10.4049/jimmunol.171.4.1684 12902466

[57] Roll P Muhammad KSchumann M Kleinart S Einsele H Dorner . *In vivo* effects of the anti-interleukin-6 receptor inhibitor tocilizumab on the b cell compartment. Arthritis rheumatism (2011) 63:1255–64. doi: 10.1002/art.30242 21305508

[58] Yang J Sakai J Siddiqui S Lee RC Ireland DDC Verthelyi D. IL-6 impairs vaccine responses in neonatal mice. Front Immunol (2018) 9:3049. doi: 10.3389/fimmu.2018.03049 30619375 PMC6307459

[59] Mitroulis I Kalafati L Bornhäuser M Hajishengallis G Chavakis T. Regulation of the bone marrow niche by inflammation. Front Immunol (2020) 11:1540. doi: 10.3389/fimmu.2020.01540 32849521 PMC7396603

[60] Valletta S Thomas A Meng Y Ren X Drissen R Sengül H. Micro-environmental sensing by bone marrow stroma identifies IL-6 and TGFβ1 as regulators of hematopoietic ageing. Nat Commun (2020) 11:4075. doi: 10.1038/s41467-020-17942-7 32796847 PMC7427787

[61] Belnoue E Tougne C Rochat AF Lambert PH Pinschewer DD Siegrist CA. Homing and adhesion patterns determine the cellular composition of the bone marrow plasma cell niche. J Immunol (Baltimore Md 1950) (2012) 188:1283–91. doi: 10.4049/jimmunol.1103169 22262758

[62] Ko EJ Lee YT Kim KH Jung YJ Lee Y Denning TL. Effects of MF59 adjuvant on induction of isotype-switched IgG antibodies and protection after immunization with T-dependent influenza virus vaccine in the absence of CD4+ T cells. J Virol (2016) 90:6976–88. doi: 10.1128/jvi.00339-16 PMC4944285 27226368

[63] Pedersen GK Wørzner K Andersen P Christensen D. Vaccine adjuvants differentially affect kinetics of antibody and germinal center responses. Front Immunol (2020) 11:579761. doi: 10.3389/fimmu.2020.579761 33072125 PMC7538648

[64] Pihlgren M Tougne C Schallert N Bozzotti P Lambert PH Siegrist CA. CpG-motifs enhance initial and sustained primary tetanus-specific antibody secreting cell responses in spleen and bone marrow, but are more effective in adult than in neonatal mice. Vaccine (2003) 21:2492–9. doi: 10.1016/S0264-410X(03)00052-5 12744883

