# Supplementary material for: A comparative study of adjuvants effects on neonatal plasma cell survival niche in bone marrow and persistence of humoral immune responses
Source: Front Immunol. 2022 Aug 3;13:904415. doi: 10.3389/fimmu.2022.904415 (PMC9381929; doi:10.3389/fimmu.2022.904415)
Supplement: Supplementary file 1 [file DataSheet_1.docx]

Supplementary Material


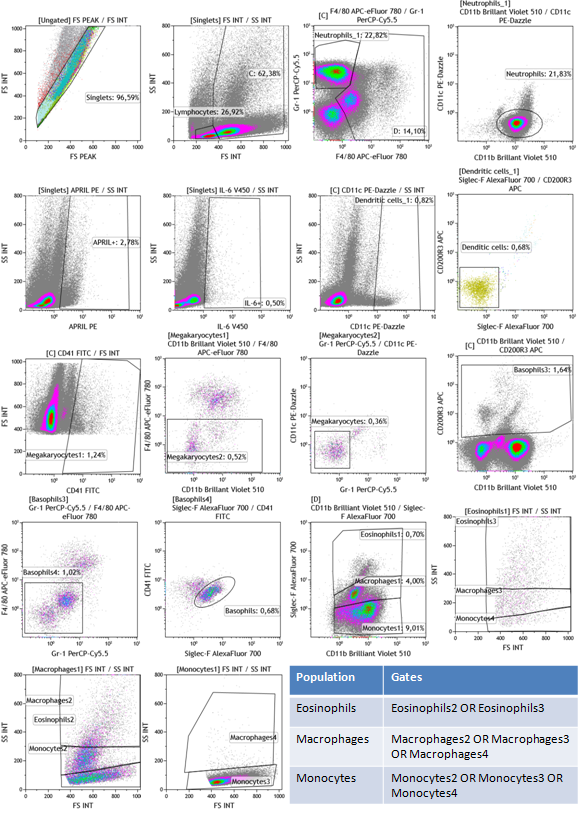


Supplementary Figure 1. Gating strategy for bone marrow accessory cells.


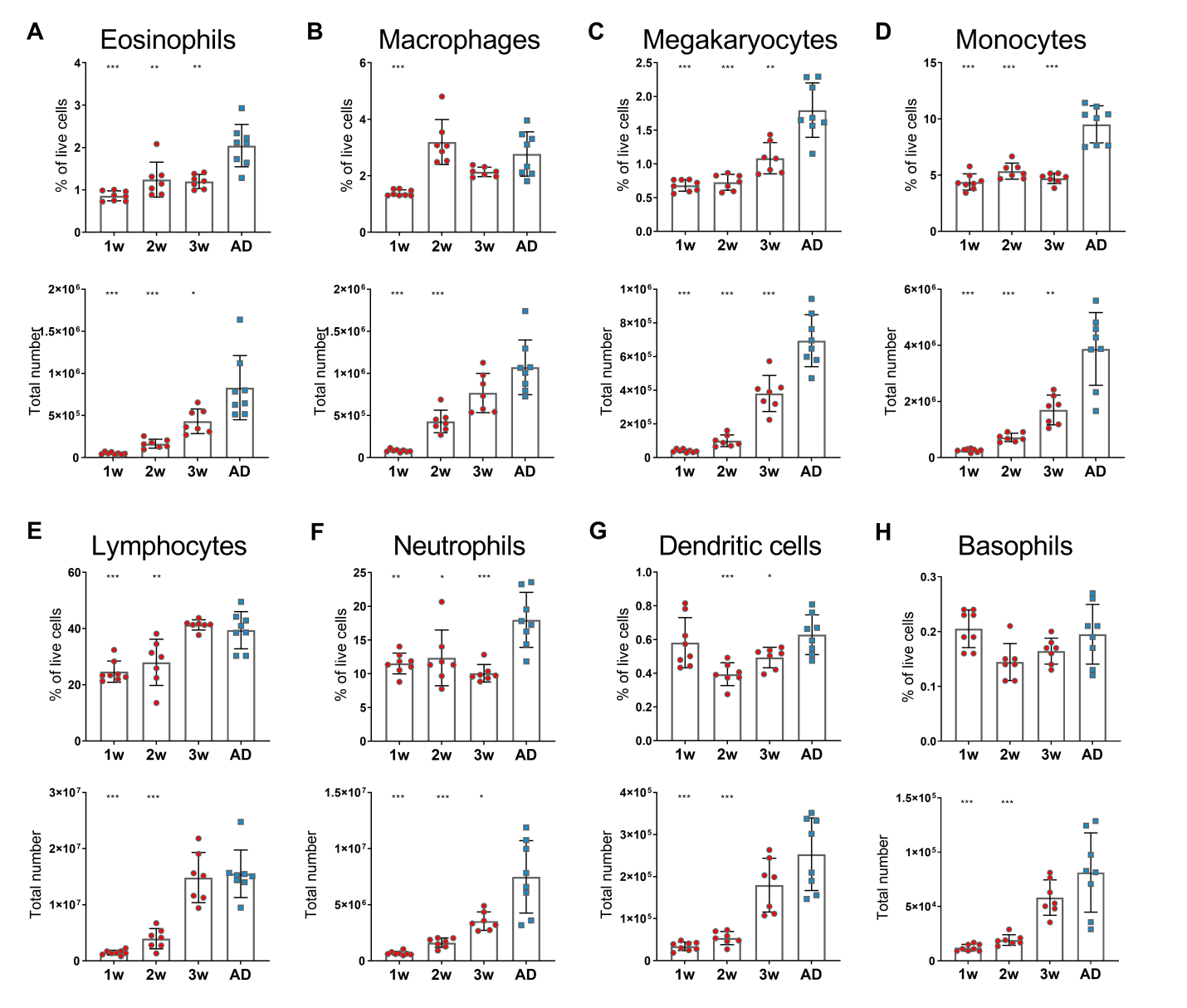


Supplementary Figure 2. Frequency and total number of eosinophils (A), macrophages (B), megakaryocytes (C), monocytes (D), lymphcoytes (E), neutrophils (F) dendritic cells (G) and basophils (H) in bone marrow assessed by flow cytometry in 1, 2, 3 weeks old and adult (AD) mice. Mann Whitney U test was used for statistical comparison where values from 1-, 2- or 3-week-old mice were compared to adult mice and *p≤0.05, **p≤0.01, ***p≤0.001.

Supplementary table 1. Average frequency and total numbers of APRIL^+^ cells in bone marrow 4, 8, 14 and 42 days following immunization of 7 days old mice with TT w/wo adjuvants LT-K63, mmCT, MF59, IC31, alum or saline.

Supplementary table 2. Average percentages of APRIL^+^ accessory cells among total APRIL^+^ cells in bone marrow 4, 8, 14 and 42 days following immunization of 7 days old mice with TT w/wo adjuvants LT-K63, mmCT, MF59, IC31, alum or saline.

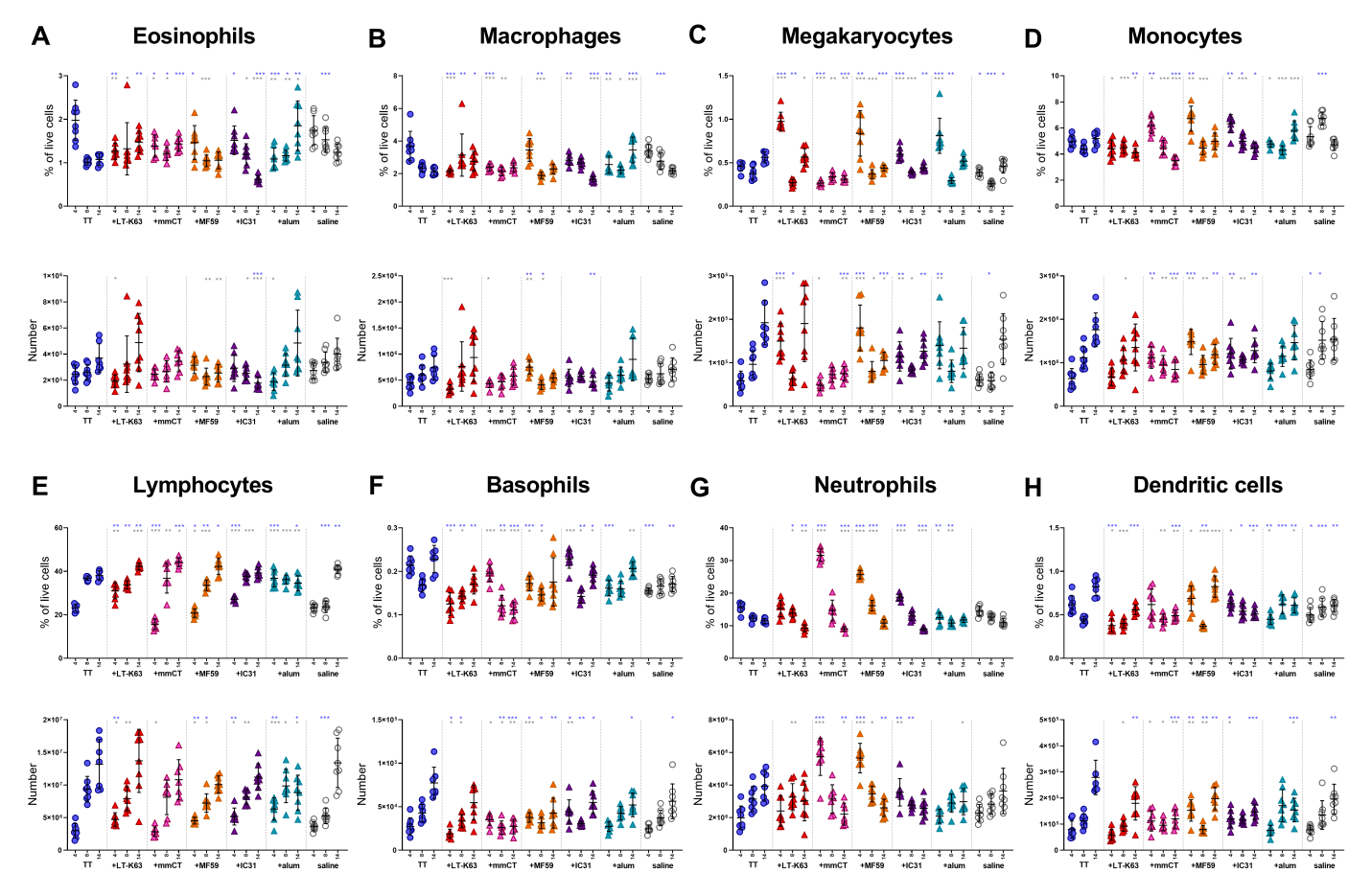


Supplementary Figure 3. Frequency and numbers of eosinophils (A), macrophages (B), megakaryocytes (C), monocytes (D), lymphocytes (E), basophils (F), neutrophils (G) and dendritic cells (H) in bone marrow 4, 8 and 14 days after neonatal immunization of with TT (blue circle) w/wo adjuvants LT-K63 (red triangle), mmCT (pink triangle), MF59 (orange triangle), IC31 (purple triangle), alum (turquoise triangle) or saline-injected mice (light grey circles) as controls. Each symbol represents one mouse and results are shown as means±SD in 8 mice per group per time point (except n=7 for TT group on day 14 and n=7 for TT+mmCT group on days 8 and 14). For statistical evaluation Mann–Whitney U-test was used. Blue stars represent p values after comparison of TT group to all other groups and grey stars represent comparisons of adjuvant groups to saline group. *p ≤ 0.05, **p ≤ 0.01, ***p ≤ 0.001.


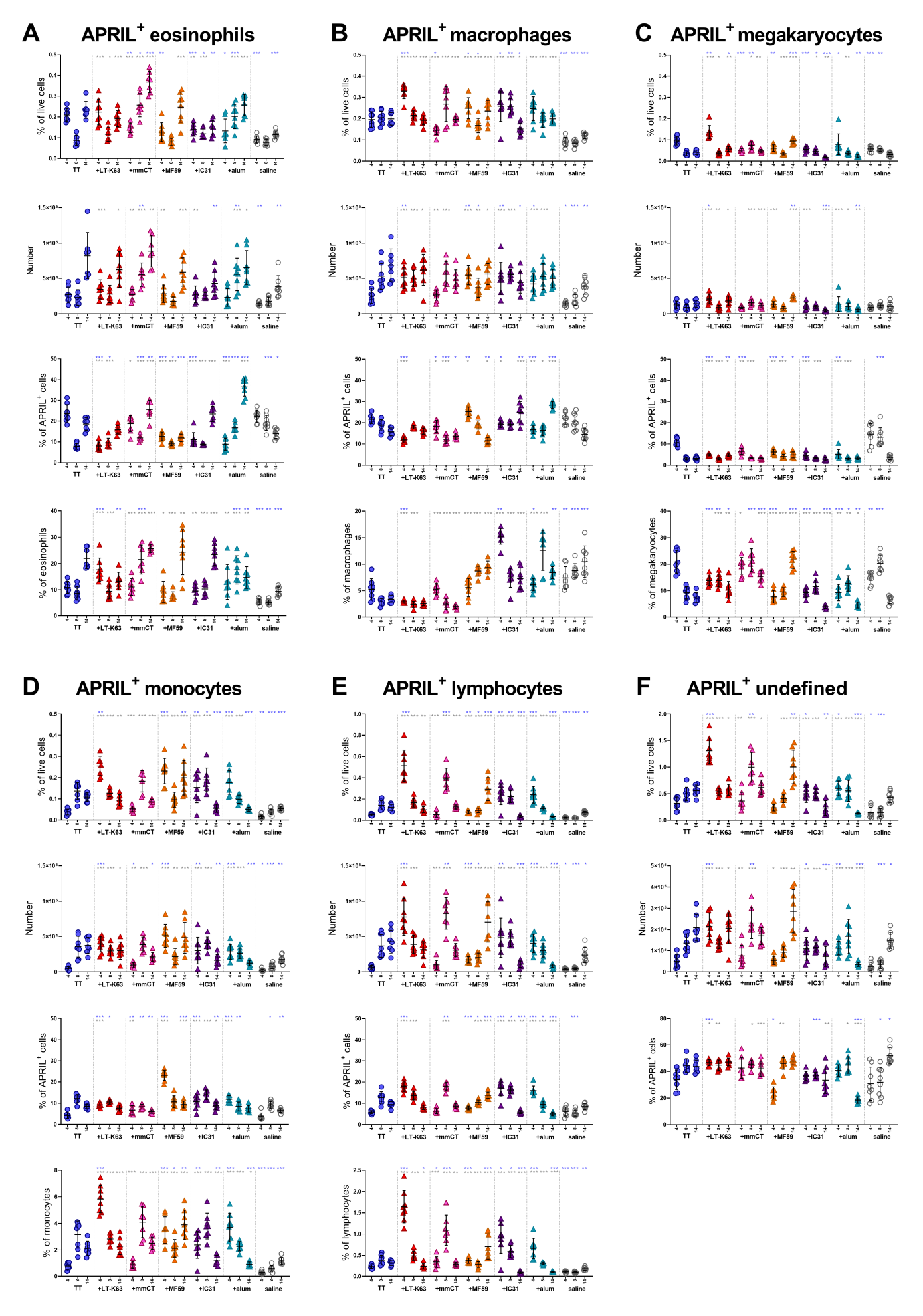


Supplementary Figure 4. Frequency, numbers, portions out of total APRIL^+^ cells and relative APRIL expression of APRIL^+^ eosinophils (A), APRIL^+^ macrophages (B), APRIL^+^ megakaryocytes (C), APRIL^+^ monocytes (D), APRIL^+^ lymphocytes (E) and frequency, number and portion of undefined APRIL^+^ cells among total APRIL^+^ cells (F) in bone marrow 4, 8 and 14 days following neonatal immunization of with TT (blue circle) w/wo adjuvants LT-K63 (red triangle), mmCT (pink triangle), MF59 (orange triangle), IC31 (purple triangle), alum (turquoise triangle) or saline-injected mice (light grey circles) as controls. Each symbol represents one mouse and results are shown as means±SD in 8 mice per group per time point (except n=7 for TT group on day 14 and n=7 for TT+mmCT group on days 8 and 14). For statistical evaluation Mann–Whitney U-test was used. Blue stars represent p values after comparison of TT group to all other groups and grey stars represent comparisons of adjuvant groups to saline group. *p ≤ 0.05, **p ≤ 0.01, ***p ≤ 0.001. Mind that y axes for APRIL^+^ lymphocytes and APRIL^+^ undefined cells are different from other cell types.


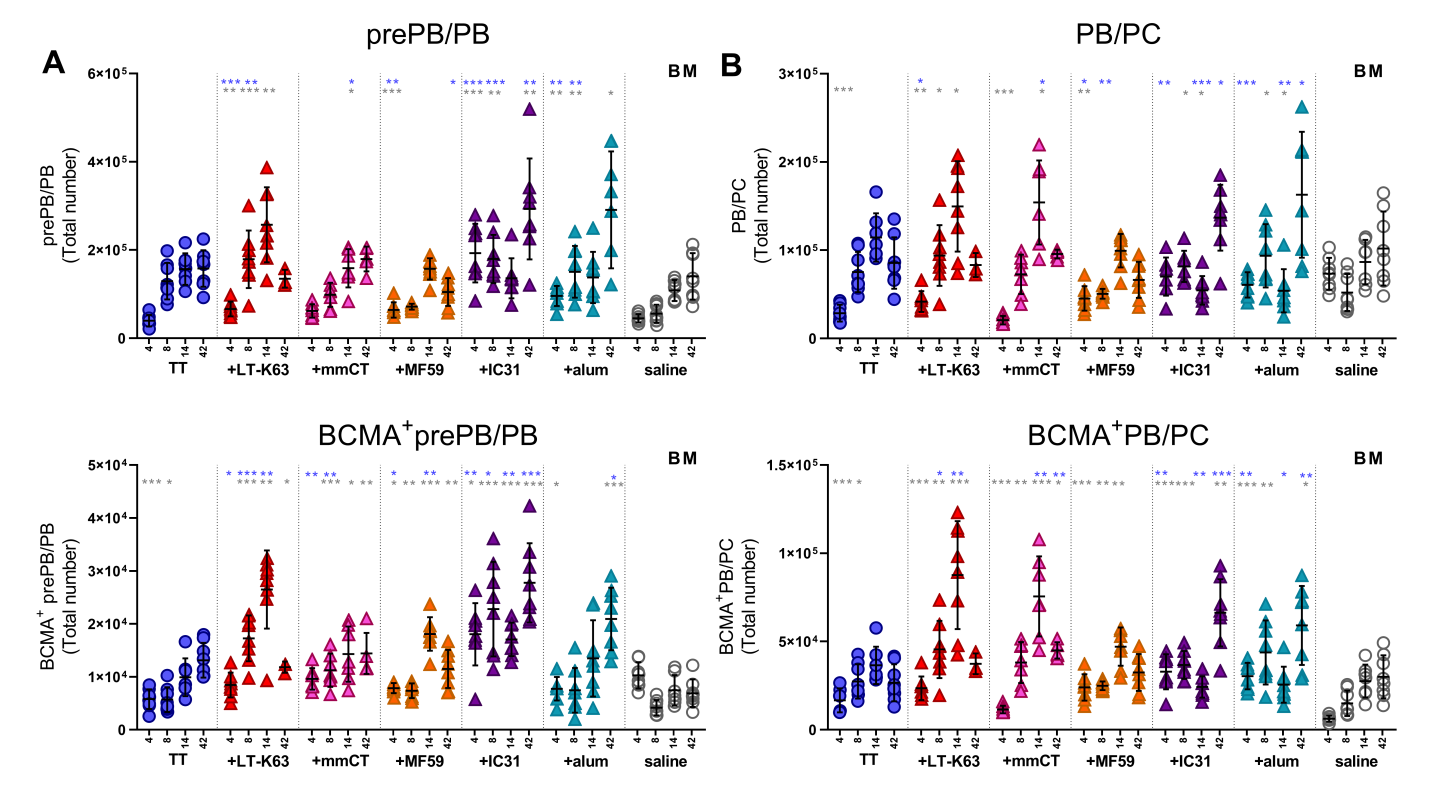


Supplementary Figure 5. Total numbers of B220^+^CD138^+^ prePB/PB and BCMA^+^B220^+^CD138^+^ prePB/PB (A), B220^+/-^CD138^high^ PB/PC and BCMA^+^B220^+/-^CD138^high^ PB/PC (B) 4, 8, 14 and 42 days after neonatal immunization with TT (blue circles) w/wo adjuvants LT-K63 (red triangle), mmCT (pink triangle), MF59 (orange triangle), IC31 (purple triangle), alum (turquoise triangle) or saline-injected mice (light grey circles) as controls. Each symbol represents one mouse and results are shown as means±SD in 8 mice per group per time point (except n=7 for TT group on day 14, n=3 for TT+LT-K63 group on day 42, n=7 for TT+mmCT group on days 8 and 14 and n=5 for TT+mmCT group on day 42). For statistical evaluation Mann–Whitney U-test was used. Blue stars represent p values after comparison of TT group to all other groups and grey stars represent comparisons of adjuvant groups to saline group. *p ≤ 0.05, **p ≤ 0.01, ***p ≤ 0.001.

Supplementary table 3. Average frequency and total numbers of IL-6^+^ cells in bone marrow 4, 8, 14 and 42 days following immunization of 7 days old mice with TT w/wo adjuvants LT-K63, mmCT, MF59, IC31, alum or saline.

Supplementary table 4. Average percentages of IL-6^+^ accessory cells among total IL-6^+^ cells in bone marrow 4, 8, 14 and 42 days following immunization of 7 days old mice with TT w/wo adjuvants LT-K63, mmCT, MF59, IC31, alum or saline.

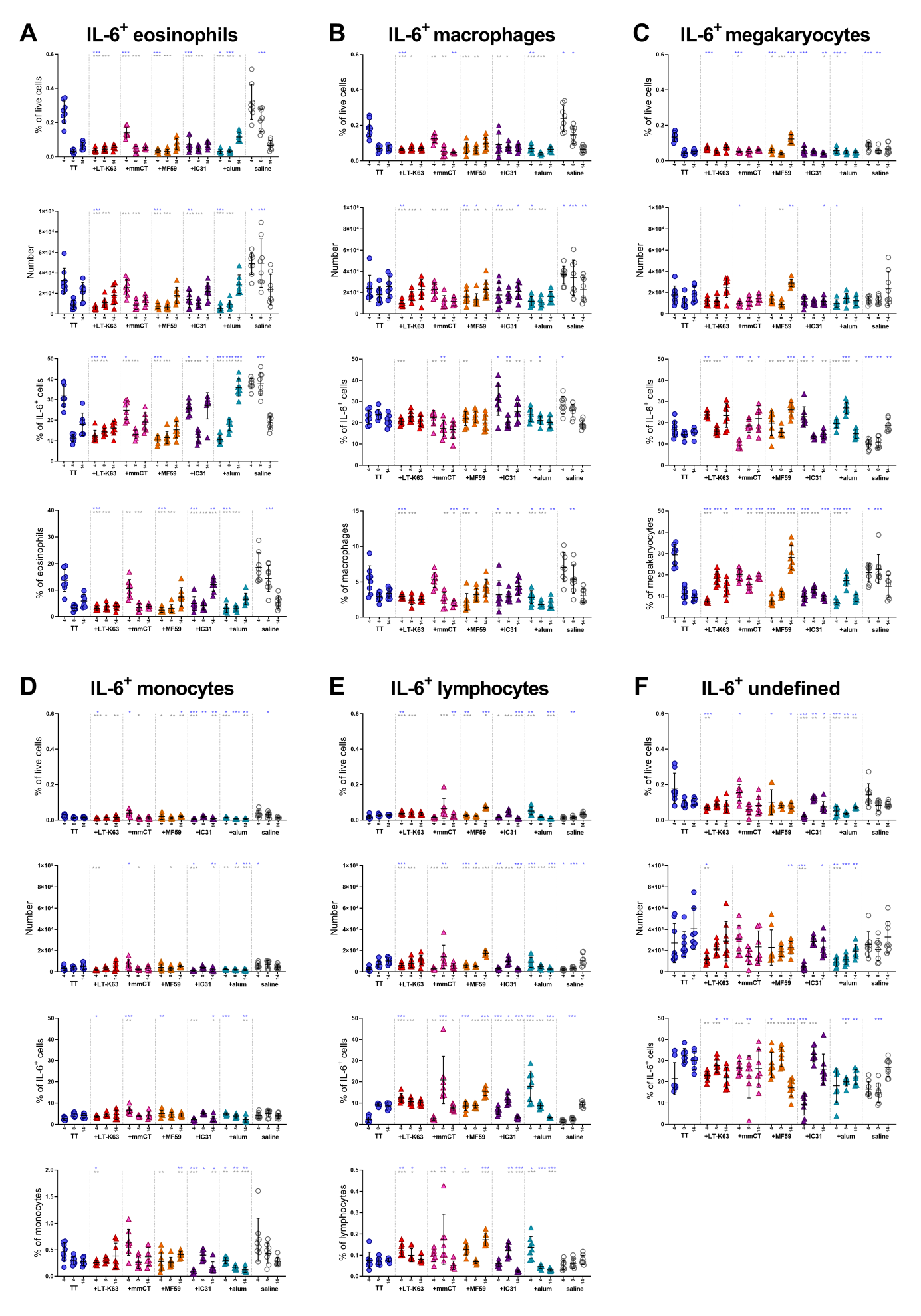

Supplementary Figure 6. Frequency, numbers, portions out of total IL-6^+^ cells and relative IL-6 expression of IL-6^+^ eosinophils (A), IL-6^+^ macrophages (B), IL-6^+^ megakaryocytes (C), IL-6^+^ monocytes (D), IL-6^+^ lymphocytes (E) and frequency, number and portion of undefined IL-6^+^ cells among total IL-6^+^ cells (F) in bone marrow 4, 8 and 14 days following neonatal immunization of with TT (blue circle) w/wo adjuvants LT-K63 (red triangle), mmCT (pink triangle), MF59 (orange triangle), IC31 (purple triangle), alum (turquoise triangle) or saline-injected mice (light grey circles) as controls. Each symbol represents one mouse and results are shown as means±SD in 8 mice per group per time point (except n=7 for TT group on day 14 and n=7 for TT+mmCT group on days 8 and 14). For statistical evaluation Mann–Whitney U-test was used. Blue stars represent p values after comparison of TT group to all other groups and grey stars represent comparisons of adjuvant groups to saline group. *p ≤ 0.05, **p ≤ 0.01, ***p ≤ 0.001.

Supplementary table 5. Average frequency and total numbers of prePB/PB and PB/PC, proportional BCMA expression of prePB/PB and PB/PC and total numbers of BCMA^+^ prePB/PB and BCMA^+^ PB/PC in bone marrow 4, 8, 14 and 42 days following immunization of 7 days old mice with TT w/wo adjuvants LT-K63, mmCT, MF59, IC31, alum or saline.

Supplementary table 6. Average frequency and total numbers of prePB/PB and PB/PC, proportional BCMA expression of prePB/PB and PB/PC and total numbers of BCMA^+^ prePB/PB and BCMA^+^ PB/PC in spleen 4, 8 and 14 days following immunization of 7 days old mice with TT w/wo adjuvants LT-K63, mmCT, MF59, IC31, alum or saline.

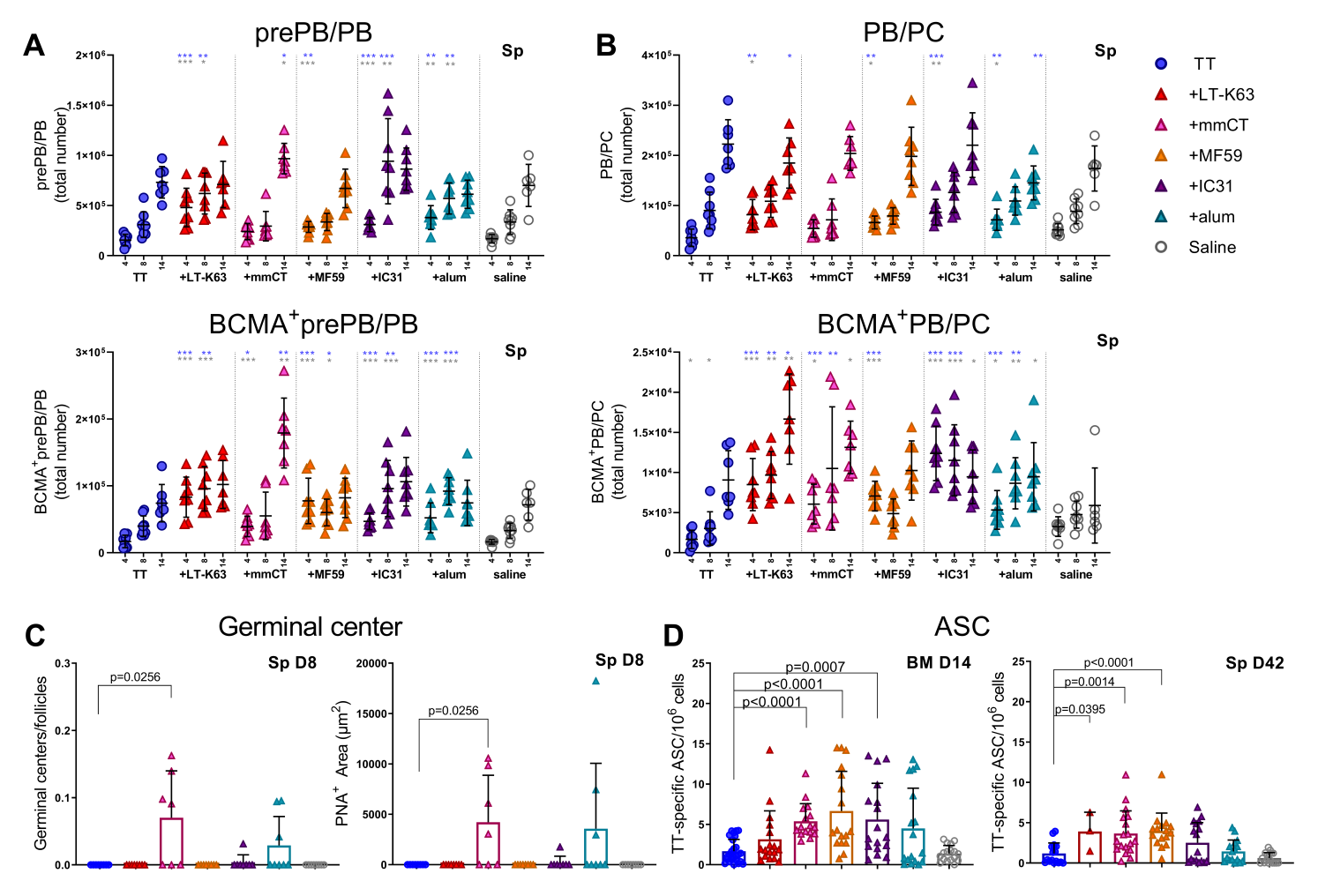


Supplementary Figure 7. Total numbers of B220^+^CD138^+^ prePB/PB and BCMA^+^B220^+^CD138^+^ prePB/PB (A), B220^+/-^CD138^high^ PB/PC and BCMA^+^B220^+/-^CD138^high^ PB/PC (B) in spleen 4, 8 and 14 days following neonatal immunization with TT (blue circles) w/wo adjuvants LT-K63 (red triangle), mmCT (pink triangle), MF59 (orange triangle), IC31 (purple triangle), alum (turquoise triangle) or saline-injected mice (light grey circles) as controls. Germinal center activation determined by fluorescent staining of spleen sections with PNA and anti-IgM 8 days after immunization of neonatal mice where PNA^+^ area represents total area of positive PNA staining per section (C). TT-specific antibody-secreting cells (ASC) in bone marrow (D, left panel) 14 days after immunization and in spleen (D, right panel) 42 days after immunization. Each symbol represents one mouse and results are shown as means±SD in 8 mice per group per time point (except n=7 for TT group on day 14, n=7 for TT+mmCT group on days 8 and 14 and n=3 for LT-K63 on day 42). For statistical evaluation Mann–Whitney U-test was used. Blue stars represent p values after comparison of TT group to all other groups and grey stars represent comparisons of adjuvant groups to saline group. *p ≤ 0.05, **p ≤ 0.01, ***p ≤ 0.001. In C-D, p values are visible on the figures.
